# Niche derived netrin-1 regulates hematopoietic stem cell dormancy via its receptor neogenin-1

**DOI:** 10.1038/s41467-020-20801-0

**Published:** 2021-01-27

**Authors:** Simon Renders, Arthur Flohr Svendsen, Jasper Panten, Nicolas Rama, Maria Maryanovich, Pia Sommerkamp, Luisa Ladel, Anna Rita Redavid, Benjamin Gibert, Seka Lazare, Benjamin Ducarouge, Katharina Schönberger, Andreas Narr, Manon Tourbez, Bertien Dethmers-Ausema, Erik Zwart, Agnes Hotz-Wagenblatt, Dachuan Zhang, Claudia Korn, Petra Zeisberger, Adriana Przybylla, Markus Sohn, Simon Mendez-Ferrer, Mathias Heikenwälder, Maik Brune, Daniel Klimmeck, Leonid Bystrykh, Paul S. Frenette, Patrick Mehlen, Gerald de Haan, Nina Cabezas-Wallscheid, Andreas Trumpp

**Affiliations:** 1 Division of Stem Cells and Cancer, German Cancer Research Center (DKFZ) and DKFZ-ZMBH Alliance, 69120 Heidelberg, Germany; 2 Heidelberg Institute for Stem Cell Technology and Experimental Medicine (HI-STEM gGmbH), 69120 Heidelberg, Germany; 3 Department of Internal Medicine V, Heidelberg University Hospital, Heidelberg, Germany; 4 Laboratory of Ageing Biology and Stem Cells, European Research Institute for the Biology of Ageing, University Medical Center Groningen, University of Groningen, Groningen, The Netherlands; 5 Faculty of Biosciences, Heidelberg University, Heidelberg, Germany; 6 Apoptosis, Cancer and Development Laboratory, Equipe labellisée “La Ligue,” LabEx DEVweCAN, Institut Convergence Rabelais, Centre de Recherche en Cancérologie de Lyon, INSERM U1052-CNRS UMR5286, Université de Lyon1, Centre Léon Bérard, 69008 Lyon, France; 7 Ruth L. and David S. Gottesman Institute for Stem Cell and Regenerative Medicine Research, Albert Einstein College of Medicine, Bronx, NY USA; 8 Department of Cell Biology, Albert Einstein College of Medicine, Bronx, NY USA; 9 Department of Medicine, Albert Einstein College of Medicine, Bronx, NY USA; 10 Max Planck Institute of Immunobiology and Epigenetics, 79108 Freiburg, Germany; 11 Core Facility Omics IT and Data Management, German Cancer Research Center (DKFZ), Heidelberg, Germany; 12 Wellcome Trust/MRC Cambridge Stem Cell Institute, University of Cambridge, Cambridge, CB2 0AH UK; 13 Department of Haematology, University of Cambridge, Cambridge, CB2 0AH UK; 14 NHS Blood and Transplant, Cambridge, CB2 0PT UK; 15 Division of Chronic Inflammation and Cancer, German Cancer Research Center Heidelberg (DKFZ), Heidelberg, Germany; 16 Department of Internal Medicine I and Clinical Chemistry, Heidelberg University Hospital, Heidelberg, Germany; 17 German Cancer Consortium (DKTK), 69120 Heidelberg, Germany

**Keywords:** Ageing, Haematopoietic stem cells, Self-renewal, Stem-cell niche

## Abstract

Haematopoietic stem cells (HSCs) are characterized by their self-renewal potential associated to dormancy. Here we identify the cell surface receptor neogenin-1 as specifically expressed in dormant HSCs. Loss of neogenin-1 initially leads to increased HSC expansion but subsequently to loss of self-renewal and premature exhaustion in vivo. Its ligand netrin-1 induces *Egr1* expression and maintains quiescence and function of cultured HSCs in a Neo1 dependent manner. Produced by arteriolar endothelial and periarteriolar stromal cells, conditional netrin-1 deletion in the bone marrow niche reduces HSC numbers, quiescence and self-renewal, while overexpression increases quiescence in vivo. Ageing associated bone marrow remodelling leads to the decline of netrin-1 expression in niches and a compensatory but reversible upregulation of neogenin-1 on HSCs. Our study suggests that niche produced netrin-1 preserves HSC quiescence and self-renewal via neogenin-1 function. Decline of netrin-1 production during ageing leads to the gradual decrease of Neo1 mediated HSC self-renewal.

## Abstract (web summary)

Haematopoietic stem cells (HSCs) are characterized by their self-renewal potential and associated dormancy. Here the authors show that niche produced netrin-1 preserves HSC quiescence and self-renewal via neogenin-1, and that decline of netrin-1 production during ageing leads to decreased Neo1 mediated HSC self-renewal.

## Introduction

Haematopoietic stem cells (HSCs) are highly quiescent and give rise to cycling multipotent progenitors (MPPs), which are in turn responsible for maintaining steady-state hematopoiesis^1–5^. Upon transplantation, HSCs harbour multilineage and serial long-term engraftment potential^6–9^. The CD34^−^ HSC compartment is heterogeneous and consists of both dormant HSCs (dHSCs) and active HSCs (aHSCs) with dHSCs showing superior serial engraftment potential^10,11^. dHSCs can be identified via label retention approaches^10–13^ or by employing *Gprc5c-GFP* reporter mice^11^. All dHSCs reside in a transcriptionally and metabolically rather inactive state and rest in the G_0_ cell cycle phase.

Upon ageing the number of immunophenotypic HSCs increases, but their self-renewal capability diminishes and a myeloid differentiation bias emerges^14–19^. Various HSC intrinsic hallmarks of ageing, such as the disruption of cellular polarity, and epigenetic instability have been identified^20–22^. Concomitantly, it has become clear that the bone marrow (BM) microenvironment undergoes remodelling upon ageing and contributes to functional decline of HSCs^23–25^. Still, the crosstalk between extrinsic niche-derived and HSC intrinsic factors mediating stem cell maintenance and quiescence, particularly in the context of ageing, remains elusive^26,27^. Based on this, we hypothesize that changes in interactions maintaining quiescence in young BM may contribute to the functional decline of HSCs.

A number of cell surface receptors, activated by niche-derived ligands such as THPO-MPL, DARC-CD82, or Histamine-H2R, have been described to directly modulate HSC behaviour^28–31^. Interestingly, some of these, including CXCR4-CXCL12 (C-X-C chemokine receptor type 4/C-X-C motif chemokine 12) and SCF-c-Kit (stem cell factor/c-Kit), also seems to play a key role during neural development^32,33^. Neogenin-1 (Neo1), a cell surface receptor first identified as a regulator of axon guidance, has been implicated in a wide variety of functions ranging from cell migration and survival to angiogenesis^34^. Its role has recently also been studied in the innate and adaptive immune systems^35–37^. It shares almost 50% amino acid homology with DCC (deleted in colorectal cancer)^38,39^. The extracellular domain of Neo1 has been described to bind members of both the “repulsive guidance molecule” (RGM-a–c) and netrin (Ntn) families^34,39^. Neo1 can modulate cytoskeletal activities and can function as a co-receptor for bone morphogenetic proteins (BMPs)^40,41^. However, the functional role of Neo1 or its ligands such as Ntn1 in HSC biology remains uncertain^1,42^. Here, we identify Ntn1–Neo1 signalling as an important regulator of HSC quiescence.

## Results

### Neo1 is specifically expressed in the most quiescent HSCs

**Fig. 1:**
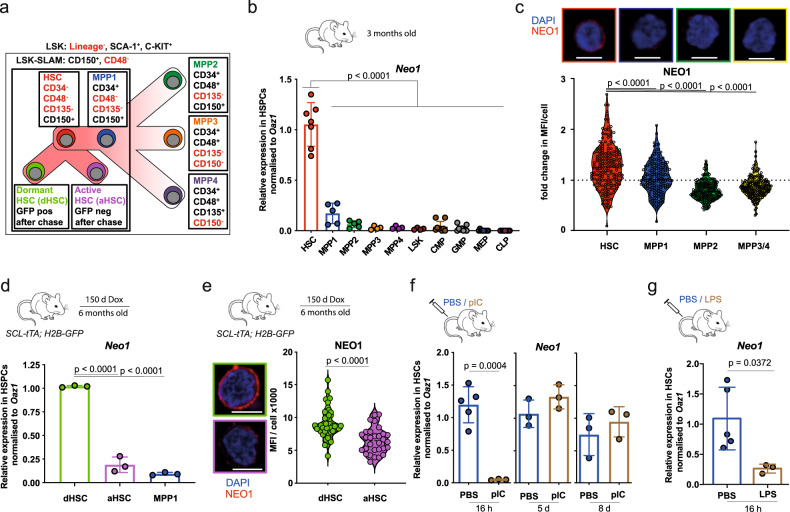
Neo1 is specifically expressed HSC and associated with quiescence. **a** Overview of hematopoietic stem and progenitor cells (HSPCs) and their immunophenotypes. **b** Relative expression of *Neo1* in HSPCs from 3-month-old mice; *n* = 4–7 (HSC-MPPs) and 9 (CMP/MEP/GMP), two independent experiments. **c** MFI of NEO1 in HSPCs from 3-month-old mice; *n* = 90 (MPP2), 118 (MPP34), 126 (MPP1) and 145 (HSC), two independent experiments. **d** Relative expression of *Neo1* in dHSC and aHSC from *SCL-tTA; H2B-GFP* mice, chase for 5 months; *n* = 3. **e** MFI of NEO1 in dHSCs and aHSCs from *SCL-tTA; H2B-GFP* mice, chase for 5 months; *n* = 30 (aHSC)–47 (dHSC). **f** Relative expression of *Neo1* in HSCs, 16 h, 5 and 7 days after PBS or poly-I:C injections; *n* = 3–5 (PBS16h). **g** Relative expression of *Neo1* in HSCs, 16 h after PBS or LPS injections; *n* = 3 (LPS)–5 (PBS). For all panels, ±SD is shown. *n* indicates biological replicates. Scale bars in IF images are 5 μm. *P* value was determined by two-tailed *t* test. Source data are provided as a Source Data file.

*Neo1* expression in HSCs has previously been reported by us and others^1,42–44^. To further characterize *Neo1* expression within the hematopoietic stem and progenitor cell (HSPC) compartment, we isolated various HSPC populations (Fig. 1a and Figure S1a) and found *Neo1* to be exclusively expressed in HSCs (Fig. 1b). This HSC-specific expression pattern of NEO1 was also apparent at the protein level (Fig. 1c and Figure S1b). NEO1 levels in HSCs were heterogeneous as ~20% of HSCs expressed particularly high levels on the surface (Fig. 1c). Next, we studied whether this subset of NEO1 high-expressing HSCs corresponds to HSCs (dHSCs) by conducting label-retaining assays using *SCL-tTA;H2B-GFP* mice^10^ (Figure S1c). After 150 days of doxycycline chase, we found *Neo1* transcripts and protein to be expressed at higher levels in dHSCs compared to aHSCs and MPP1s, suggesting that *Neo1* is associated with dormancy (Fig. 1d, e). As expected, dHSCs specifically expressed the dHSC marker *Gprc5c*^11^ (Figure S1d). To independently validate increased *Neo1* expression in dHSCs, we employed *Gprc5c-GFP* reporter mice and isolated dormant GFP^pos^ and active GFP^neg^ HSCs (Figure S1e). In agreement, we found higher Neo1 RNA and protein levels in Gprc5c-GFP^pos^ vs. Gprc5c-GFP^neg^ HSCs (Figure S1f, g). As HSCs are a highly quiescent population during steady state, we next addressed whether *Neo1* levels not only rapidly diminished during hematopoietic differentiation, but also upon HSC activation. Therefore, we treated mice with either poly-I:C (pIC) mimicking viral, or lipopolysaccharide (LPS) mimicking bacterial infection^45,46^. HSCs showed a robust, reversible loss of *Neo1* expression in response to either stimulus (Fig. 1f, g). Collectively, these data strongly link *Neo1* expression to dormancy in HSCs.

### *Neo1*-mutant mice reveal a competitive advantage upon transplantation

**Fig. 2:**
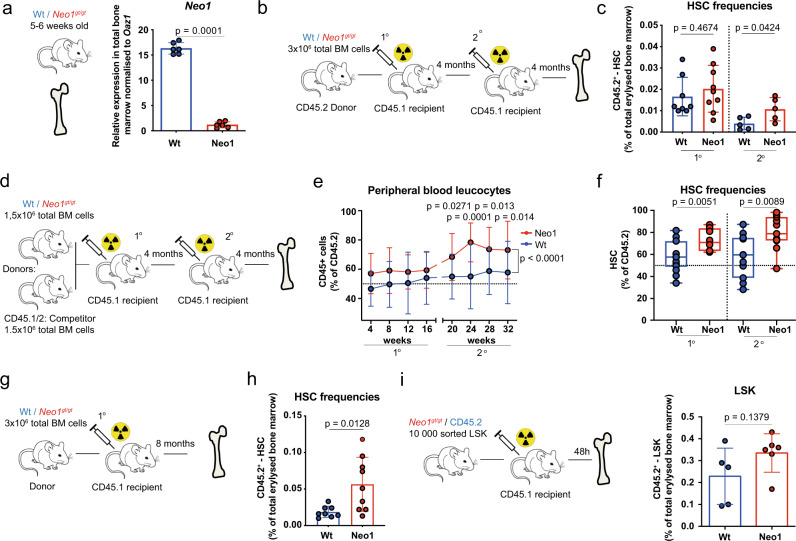
Mutant Neo1 causes an initial HSC expansion. **a** Relative expression of *Neo1* in the total bone marrow of Wt and *Neo1*^*gt/gt*^ mice; *n* = 6, three independent experiments. **b** Workflow: generation of full chimeras. **c** Absolute frequencies of bone marrow CD45.2^+^ HSCs in full Wt and *Neo1*^*gt/gt*^ chimeras 4 months after first and second transplantation; *n* = 5 (2nd Tx)–8 (Ctrl 1st Tx) and 9 (Neo1 1st TX), two independent experiments. **d** Workflow: competitive transplantations. **e** Peripheral blood CD45.2^+^ chimerism during 1° and 2° competitive transplantations of Wt and *Neo1*^*gt/gt*^ bone marrow; *n* = 13–17 (for exact *n*/timepoint please see Source data file), three independent experiments, Analysis with two-way ANOVA, multiple comparison with LSD Fisher’s test. **f** CD45.2^+^ chimerism of HSCs at endpoints of 1° and 2° competitive transplantations of Wt and *Neo1*^*gt//gt*^ bone marrow; *n* = 11 (2nd TX), 12 (Ctrl 1st Tx), 14(Neo1 1st TX), three independent experiments. Whiskers are min–max, box is 25–75th percentile and line is mean. **g** Workflow: full chimeras studied in (**h**). **h** Absolute frequencies of bone marrow CD45.2^+^ HSCs in full Wt and *Neo1*^*gt//gt*^ chimeras after 8 months; *n* = 8 (Ctrl)–9 (Neo1), three independent experiments. **i** Workflow: Homing assay in (**j**). **j** Absolute frequencies of CD45.2^+^ bone marrow LSK cells 48 h after transplantation of 10,000 sorted Wt and *Neo1*^*gt//gt*^ LSK; *n* = 5 (Ctrl)–6 (Neo1). For all panels, ±SD is shown. *n* indicates biological replicates. *P* value was determined by two-tailed *t* test unless stated otherwise. Source data are provided as a Source Data file.

Considering the HSC-specific expression pattern of *Neo1*, we set out to study the function of Neo1 in the hematopoietic system. Unfortunately, in our hands, no commercial antibody allowed the robust and reproducible isolation of viable Neo1^+^ cells by flow cytometry when using *Neo1*-mutant cells as controls^42^. Thus, we employed a *Neo1* gene-trapped mouse model to genetically address the functional role of Neo1 in HSC biology (*Neo1*^*gt/gt*^)^38,47,48^. Although *Neo1* expression in the BM of mutant mice was diminished by >90% (Fig. 2a), the hematopoietic compartment did not exhibit altered HSPC or mature cell frequencies in 5–6-week-old animals (Figure S1h). To analyse *Neo1*-deficient hematopoiesis, we performed reconstitution analysis with BM cells derived from 5- to 6-week-old *Neo1*-mutant animals (Fig. 2b). First, we non-competitively transplanted total BM derived from *Neo1*-mutant or control littermates (CD45.2) into CD45.1 recipients and assessed HSC numbers 4 months after primary or secondary transplantation (Fig. 2c). We observed that the frequency of HSCs, while similar at 4 months after transplantation, increased in *Neo1*-mutant chimeras upon secondary transplantation. To further investigate this expansion of HSCs, we performed competitive transplantations of *Neo1*-mutant or control BM cells (Fig. 2d). We found that *Neo1*-mutant BM cells showed a competitive advantage compared to control counterparts as evident by peripheral blood leucocyte contribution in secondary recipients and in BM HSC contribution in primary and secondary transplantations (Fig. 2e, f). As HSC frequencies in both transplantation assays increased over time, we also investigated primary chimeras 8 months after transplantation and again found an increase in HSC numbers in *Neo1*-mutant chimeras (Fig. 2g, h). We observed no difference in HSC homing (Fig. 2i, j), suggesting that self-renewal and output of *Neo1*-mutant HSCs are altered.

### Aged *Neo1*-mutant HSCs display features of premature exhaustion

**Fig. 3:**
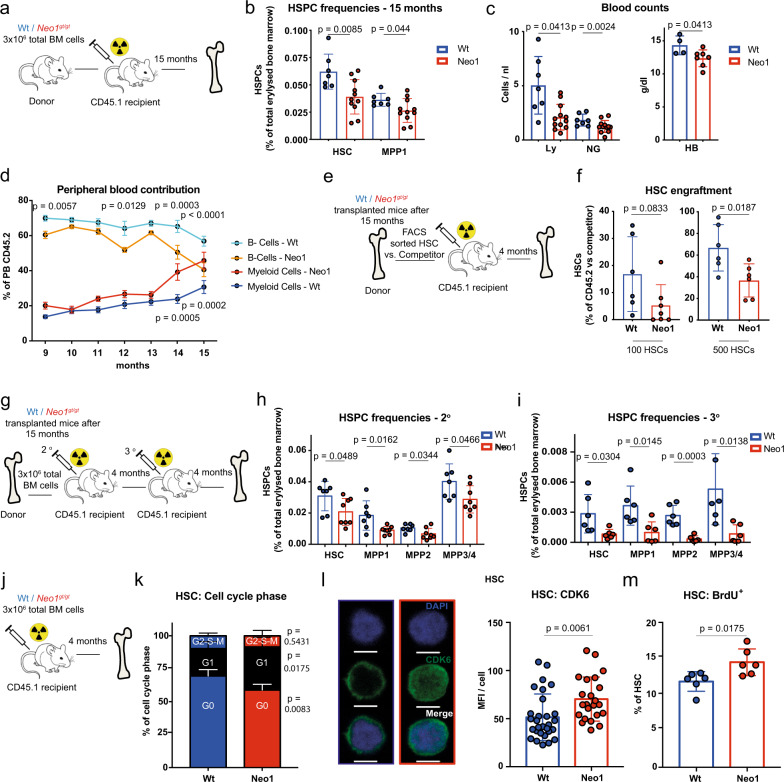
Mutant Neo1 causes premature HSC exhaustion. **a** Workflow: aged chimeras, analysed in (**b**–**d**). **b** Absolute frequencies of bone marrow CD45.2^+^ HSPCs in full Wt and *Neo1*^*gt//gt*^ chimeras after 15 months; *n* = 7 (Ctrl)–11 (Neo1), two independent experiments. **c** Absolute blood counts of full Wt and *Neo1*^*gt//gt*^ chimeras after 15 months; *n* = 7 (Ctrl)–11 (Neo1), two independent experiments, for HB: 4 (Ctrl)–7 (Neo1). **d** Frequencies of B cells and myeloid cells of C45.2^+^ cells in peripheral blood of Wt and *Neo1*^*gt//gt*^ chimeras after 15 months; *n* = 5–13 (for exact *n*/timepoint please see Source data file), two independent experiments. Analysis with two-way-ANOVA, multiple comparisons with LSD Fisher’s test. **e** Workflow: assessment of HSC potency derived from 15 months (aged) chimeras. **f** Frequency of CD45.2^+^ vs. competitor HSCs 16 weeks transplantation of 100 or 500 HSCs from of aged Wt and *Neo1*^*gt//gt*^ chimeras; *n* = 6 (Ctrl + 500 HSC Neo1)–7(100 HSC, Neo1), two independent experiments. **g** Workflow: secondary and tertiary transplantations of 15 months (aged) chimeras. **h** Absolute frequencies of bone marrow CD45.2^+^ HSPCs in 2° transplantations of aged Wt and *Neo1*^*gt//gt*^ chimeras after 4 months; *n* = 7 (Ctrl)–8 (Neo1), two independent experiments. **i** Absolute frequencies of bone marrow CD45.2^+^ HSPCs in 3° transplantations of aged Wt and *Neo1*^*gt//gt*^ chimeras after 4 months; *n* = 6, two independent experiments. **j** Workflow: generation of full chimeras used in (**k**–**m**). **k** Cell cycle phase of CD45.2^+^ HSCs derived from Wt and *Neo1*^*gt//gt*^ chimeras after 4 months; *n* = 4 (Ctrl)–6 (Neo1), two independent experiments. **l** MFI of CDK6 in CD45.2^+^ HSC derived from Wt and *Neo1*^*gt//gt*^ chimeras after 4 months; *n* = 23 (Neo1)–29 (Ctrl). **m** Frequency of BrdU^+^ CD45.2^+^ HSC derived from Wt and *Neo1*^*gt//gt*^ chimeras after 4 months, 48 h post BrdU injection; *n* = 6, two independent experiments. For all panels, ±SD is shown. *n* indicates biological replicates. *P* value was determined by two-tailed *t* test unless stated otherwise. Scale bars in IF images are 5 μm. Source data are provided as a Source Data file.

Next, we addressed whether the HSC expansion observed in *Neo1*-mutant chimeras would lead to malignant transformation or HSC exhaustion over time (Fig. 3a). Interestingly, 15 months after the generation of primary chimeras, the initial expansion of the *Neo1*-mutant HSC pool reverted and both HSC and MPP1 frequencies decreased (Fig. 3b). When we compared absolute blood counts in aged *Neo1*-mutant chimeras to controls, we found reduced absolute lymphocyte and neutrophil counts, as well as reduced haemoglobin levels indicative of hematopoietic malfunction (Fig. 3c). As expected, chimeras displayed increased myeloid differentiation upon ageing, and this effect was exacerbated in *Neo1*-mutant chimeras over time (Fig. 3d). To address whether this decline in mature cell output was caused by an HSC defect, we re-transplanted 100 CD45.2^+^ HSCs derived from either aged *Neo1*-mutant or control chimeras (Fig. 3e). Four months after transplantation, *Neo1*-mutant HSCs had generated significantly less progeny then controls (Fig. 3f). To validate functional exhaustion, we re-transplanted BM of aged chimeras into secondary and tertiary recipients (Fig. 3g). In these mice, aged *Neo1*-mutant BM exhibited a pronounced failure to engraft and depletion of HSCs and all MPP populations was observed, suggesting that the original *Neo1*-mutant HSCs from the aged chimeras had a decreased self-renewal potential (Fig. 3h, i). Meanwhile, we observed no increase in malignancies arising in *Neo1*-mutant chimeras. Next, we analysed cell cycle behaviour of *Neo1*-mutant HSCs. We found less HSCs residing in the G0 phase in 4–5-week-old *Neo1*-mutant mice compared to their control littermates (Figure S1i). This decrease in G0-HSCs was also apparent in full chimeras both 4 and 8 months after transplantation (Fig. 3j, k and Figure S1j) and *Neo1*-mutant HSCs expressed higher levels of the cell cycle activation marker CDK6. In addition, increased incorporation of bromodeoxyuridine (BrdU) above the expected injection-induced activation was observed in *Neo1*-mutant HSCs (Fig. 3l, m and Figure S1k). Altogether, *Neo1*-mutant HSCs harbour diminished long-term repopulation potential, associated with a loss of quiescence and increased proliferation.

### Molecular signatures of activation and HSC dysfunction are enriched in *Neo1-*mutant HSCs

**Fig. 4:**
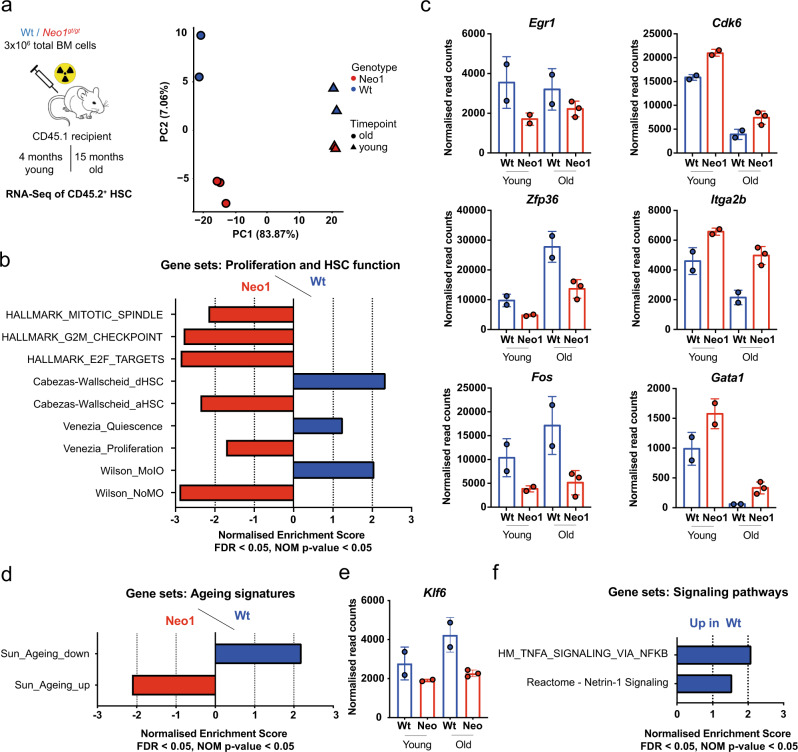
Neo1-mutant HSCs reveal a loss of quiescence and potency signatures. **a**, Left: workflow for RNA-seq of CD45.2^+^ HSCs from Wt and *Neo1*^*gt//gt*^ chimeras after 4 and 15 months. Right: sparse PCA; *n* = 2 (WT old/young, Neo1 young)–3 (Neo1 old). **b** GSEA for cell cycle and HSC potency of Wt vs. *Neo1*^*gt//gt*^ HSCs. FDR < 0.05, NOM *p* value <0.05. **c** Normalized read counts of DEG in HSCs from young and old Wt and *Neo1*^*gt//gt*^ chimeras, *n* = 4 (Ctrl)–5 (Neo1). **d** GSEA for HSC ageing signatures in Wt vs. *Neo1*^*gt//gt*^ HSCs. FDR < 0.05, NOM *p* value <0.05. **e** Normalized read counts of *Klf6* in HSCs from young and old Wt and *Neo1*^*gt//gt*^ chimeras, *n* = 4 (Ctrl)–5 (Neo1). **f** GSEA for signalling pathways in Wt vs. *Neo1*^*gt//gt*^ HSCs. FDR < 0.05, NOM *p* value <0.05^.^ For all panels, ±SD is shown. *n* indicates biological replicates. Scale bars in IF images are 4 μm. *P* value was determined by two-tailed *t* test unless stated otherwise. Source data are provided as a Source Data file.

To understand the molecular basis for the disruption of long-term self-renewal caused by loss of Neo1, we performed RNA-sequencing (RNA-seq) analysis of *Neo1*^*gt/gt*^ and wild-type (Wt) CD45.2^+^ HSCs 4 months (expanding *Neo1*-mutant HSCs) and 15 months (exhausted *Neo1*-mutant HSCs) after transplantation (Fig. 4a and Figure S2a). The principal component analysis showed the main mode of transcriptional variation to be attributable to age. The molecular consequences of mutant *Neo1* were recapitulated by PC-2 and the difference increased upon ageing (Fig. 4a and Figure S2b). As expected, *Neo1* expression itself was diminished in *Neo1*-mutant HSCs, but interestingly strongly upregulated in aged compared to young Wt HSCs (Figure S2c). Analysis of shared functional differences between young and old *Neo1*-mutant HSCs compared to controls using Gene Set Enrichment Analysis (GSEA), we revealed cell cycle-associated gene sets like *Hallmark(HM)_Mitotic_Spindle* and *HM_G2M_Checkpoint to be* enriched in *Neo1* mutants (Fig. 4b) validating the functional data. This pattern of increased activation in *Neo1*-mutant HSCs was also observed employing HSC-specific cell cycle signatures^49^ (Fig. 4b). In line with these data, the signature for *aHSCs* was enriched in *Neo1*-mutant HSCs, in turn the signature for *dHSCs* was enriched in Wt HSCs^11^ (Fig. 4b). Reflecting the observed functional deficits of *Neo1*-mutant HSCs, the *MoIO*^50^ signature associated with superior HSC function was overrepresented in Wt HSCs, while the *NoMO* signature^50^, enriching for less quiescent, functionally inferior HSCs were enriched in *Neo1*-mutant HSCs (Fig. 4b). Analysis of differentially expressed genes (DEGs) identified genes associated with differentiation such as *Itga2b* and *Gata1*^50–52^, as well as cell cycle regulators such as *Cdk6*^53^ (Fig. 4c and Figure S2f) or *Mki67* (Figure S2d) to be upregulated in *Neo1*-mutant HSCs. In contrast, genes known to regulate HSC self-renewal or quiescence, such as *Egr1*^54,55^, *Zfp36*^56^ and c-*Fos*^57^ were downregulated (Fig. 4c and Figure S2f). Interestingly, Cdk6 has been shown to suppress *Egr1* expression during HSC activation, which was suggested to promote HSC quiescence based on genetic data and thus is a likely downstream target of Neo1^54^. No other Ntn1 receptors were differentially expressed (Figure S2e). Therefore, the molecular data support the functional findings by revealing footprints of both loss of quiescence and diminished expression of HSC self-renewal related genes in *Neo1*-mutant HSCs. In addition, we found that HSC ageing signatures^20^ were enriched in *Neo1*-mutant HSCs reflecting the observed functional decline (Fig. 4d). In line, *Klf6*, which has been proposed to maintain features of young HSCs in human, was downregulated in *Neo1*-mutant HSCs^58^ (Fig. 4e and Figure S2f). Finally, we report gene sets associated with nuclear factor-κB (NF-κB) signalling, as well as signalling of the NEO1 ligand netrin-1 (Ntn1) to be depleted in *Neo1*-mutant HSCs, suggesting that these signalling pathways may be downstream of NEO1 activation (Fig. 4f).

Interestingly, when we tested enrichment for the *Reactome_Netrin-1_Signalling* gene set on RNA-seq data of a recent study of HSPC^1^, it was enriched in HSCs compared to all MPP populations, suggesting that Ntn1 signalling is physiologically active in homeostatic HSCs (Figure S2g). In summary, we discover molecular features of both loss of quiescence and loss of self-renewal in *Neo*1-mutant HSCs, paralleling functional results.

### NTN1 maintains HSC engraftment potential and quiescence via NEO1 signalling

**Fig. 5:**
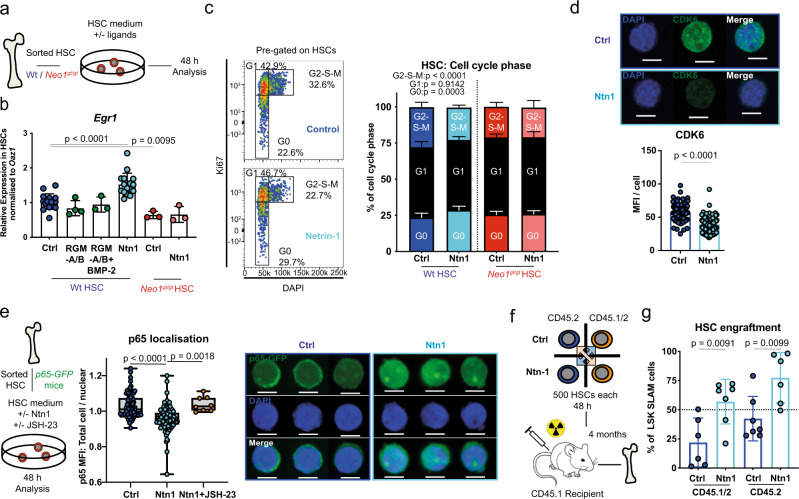
Ntn1 preserves HSC quiescence and engraftment potential in vitro via Neo1. **a** Workflow: In vitro stimulation of sorted HSCs used in (**b**–**d**), analysis after 48 h. **b** Relative expression of *Egr1* in Wt HSCs; *n* = 3 (other), 4 (RGM-a + b), 16 (Ctrl/Neo1), for ctrl/Ntn1, four independent experiments. **c** Representative cell cycle plots pre-gated on HSCs and quantification with or without Ntn1 treatment; *n* = 3 (Neo1), 11 (Wt-Ctrl), 12 (Wt-Ntn1), three independent experiments for ctrl HSC. **d** MFI of CDK6 in Wt HSCs 48 h after Ntn1 treatment, quantification of MFI per cell; *n* = 114 (Ctrl) and 134 (Ntn1). **e** Workflow: representative images and quantification of total cell/nuclear MFI of p65-GFP HSC 48 h after treatment with Ntn1 or Ntn1 + JSH-23; *n* = 8 (JSH-23), 78 (Ctrl), 91 (Ntn1), two independent experiments. **f** Workflow: competitive transplantation of Ntn1 stimulated CD45.2 and CD45.1/2 HSCs. **g** Chimerism of bone marrow LSK-SLAM cells 4 months after competitive transplantation of Control vs. Ntn1-treated HSCs; *n* = 6 (CD45.1/2), 7 (CD45.2), two independent experiments. For all panels, ±SD is shown. *n* indicates biological replicates. Scale bars in IF images are 4 μm. *P* value was determined by two-tailed *t* test unless stated otherwise. Source data are provided as a Source Data file.

Next, we assessed whether the NEO1 ligands NTN1, RGM-a and RGM-b alone or in combination with their co-ligand BMP-2 were able to affect HSC behaviour. Because neither *RGM*s and *Ntn1* nor additional Ntn1 receptors were expressed in HSCs (Figure S2h)^1^, we sorted and cultured HSCs in the presence of NTN1, RGM-A and RGM-B with or without BMP-2 (Fig. 5a). To assess active NEO1 signalling, we monitored *Egr1* expression, which was downregulated in *Neo1*-mutant HSCs (Fig. 5b). After 48 h of stimulation, only NTN1, but none of the other ligands, induced expression of *Egr1* (Fig. 5b). This induction was absent in *Neo1*-mutant HSCs (Fig. 5b). In addition, we detected a Neo1-dependent decrease in G2–S–M and an increase in G0-phase HSCs as well as diminished CDK6 protein levels (Fig. 5c, d), paralleling the data from *Neo1*-mutant HSCs in vivo (Fig. 4c, g). We further confirmed the induction of quiescence by NTN1 with HSCs isolated from *FUCCI*^59^ and *c-Myc*-*GFP* mice^60^ reporter mice (Figure S2i, j). Gene sets associated with NF-κB signalling were downregulated in *Neo1*-mutant HSCs. Since NF-κB is essential for HSC maintenance and known to protect HSCs from premature differentiation upon stress^61^, we hypothesized that NTN1 may induce NF-κB signalling. To test this hypothesis, we isolated HSCs from *p65-GFP* mice, cultured them +/− NTN1 or +/− the p65 nuclear translocation inhibitor JSH-23 (Fig. 5e). We observed increased nuclear p65 levels upon NTN1 treatment, which was blocked by JSH-23 (Fig. 5e), suggesting that NTN1 maintains the canonical NF-κB pathway. We next assessed whether in vitro NTN1 stimulation translates into improved HSC engraftment in vivo. For this purpose, we stimulated 500 HSCs derived from either CD45.2 or CD45.1/2 mice with or without Ntn1 for 48 h, mixed treated with untreated congenically distinct HSCs and transplanted them into lethally irradiated recipients (Fig. 5f). Four months after transplantation, we found increased engraftment of HSCs cultured with NTN1 in the BM, independent of genotype (Fig. 5g). This showed that ex vivo treatment with NTN1 robustly improved the in vivo function of cultured HSCs. This effect of NTN1 was dependent on the presence of NEO1 since it was absent in *Neo1*-mutant HSCs (Figure S2k). Collectively, these data suggest that the NTN1–NEO1 axis preserves NF-κB activity, quiescence and in vivo function of cultured HSCs.

### Conditional *Ntn1* deletion depletes HSCs and leads to activation and differentiation in vivo

**Fig. 6:**
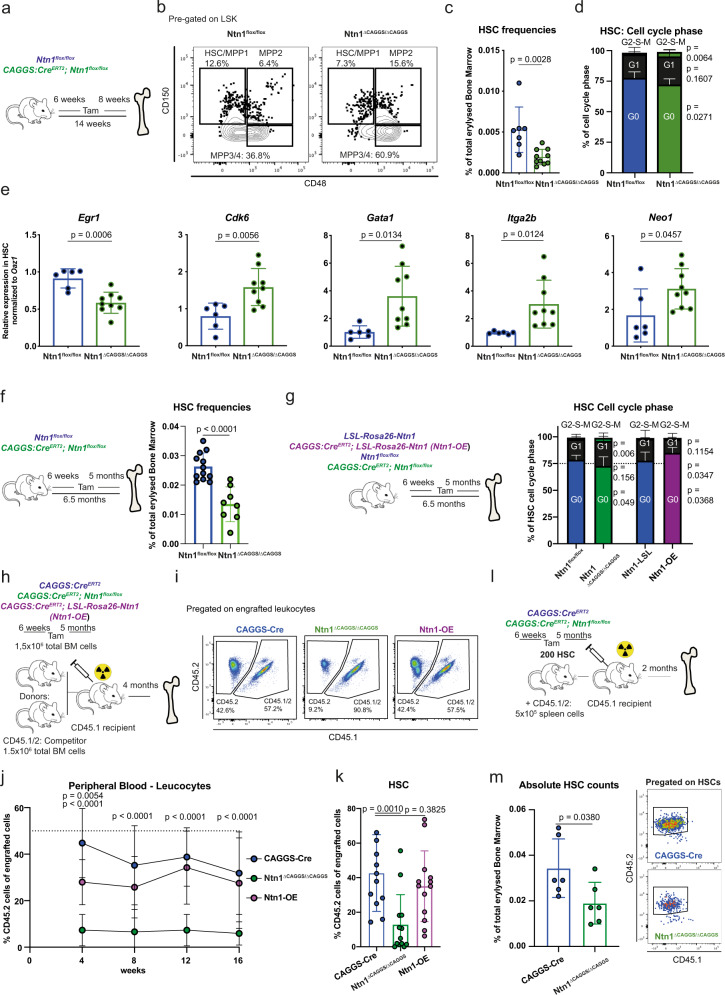
In vivo *Ntn*1 deletion depletes HSC and Ntn1 overexpression increases HSC quiescence. **a** Workflow: analysis of *Ntn1*^*flox/flox*^ and *CAGGS:Cre*^*ERT2*^*; Ntn1*^*flox/flox*^ mice 8 weeks after Cre induction for (**b**–**e**). **b** Representative flow cytometry plots of the LSK population of *Ntn1*^*flox/flox*^ and *Ntn1*^*ΔCAGGS/ΔCAGGS*^ mice. **c** Frequencies of bone marrow HSCs in *Ntn1*^*flox/flox*^ and *Ntn1*^*ΔCAGGS/ΔCAGGS*^ mice; *n* = 7 (flox)–10 (∆CAGGS), two independent experiments. **d** Cell cycle phase of HSCs derived from *Ntn1*^*flox/flox*^ and *Ntn1*^*ΔCAGGS/ΔCAGGS*^ mice; *n* = 8 (flox) and 10 (∆CAGGS), two independent experiments. **e** Relative expression of quiescence and activation related genes in HSCs derived from *Ntn1*^*flox/flox*^ and *Ntn1*^*ΔCAGGS/ΔCAGGS*^ mice; *n* = 6 (flox)–9 (∆CAGGS), two independent experiments. **f** Frequencies of bone marrow HSCs in *Ntn1*^*flox/flox*^ and *Ntn1*^*ΔCAGGS/ΔCAGGS*^ mice 5 months after Cre induction; *n* = 8 (∆CAGGS) and 12 (flox), three independent experiments. **g** Cell cycle phase of HSCs derived from *Ntn1*^*+/LSL-Rosa26-Ntn1*^ and *Ntn1-OE* mice; *n* = 8 (∆CAGGS) and 12 (flox), three independent experiments. **h** Workflow: competitive transplantation of *CAGGS:Cre*^*ERT2*^, *Ntn1*^*ΔCAGGS/ΔCAGGS*^ and Ntn1-OE mice 5 months after Cre induction, analysed in (**i**–**k**). **i** Representative FACS plots of peripheral blood leucocytes pre-gated on CD45^+^ cells at 16 weeks after transplantation. **j** Peripheral blood CD45.2^+^ chimerism during competitive transplantations; *n* = 13 (OE)–14 (Cre/∆CAGGS), two independent experiments. Analysis was done with two-way-ANOVA, multiple comparison with LSD Fisher’s test. **k** Bone marrow HSC CD45.2^+^ chimerism after 16 weeks of competitive transplantation; *n* = 12 (Cre)–13 (∆CAGGS/OE), two independent experiments. **l** Workflow: transplantation of 200 HSCs sorted from *CAGGS:Cre*^*ERT2*^ and *Ntn1*^*ΔCAGGS/ΔCAGGS*^ mice at 5 months after Cre induction. **m** Frequencies of bone marrow HSCs 8 weeks transplantation; *n* = 6. For all panels, ±SD is shown. *n* indicates biological replicates. *P* value was determined by two-tailed *t* test unless stated otherwise. Source data are provided as a Source Data file.

Next, we addressed the role of Ntn1 in hematopoiesis in vivo. Mice homozygous for an Ntn1-null allele (*Ntn1*^*β-geo/*^^*β*^^*-geo*^) die perinatally due to defects in cerebral development^62^ and heterozygous mice display no hematopoietic phenotype (Figure S3a). Therefore, we generated *CAGGS:Cre*^*ERT2*^; *Ntn1*^*flox/flox*^ mice^63,64^, which allows tamoxifen (Tam)-inducible ubiquitous deletion of *Ntn1* (Figure S3h). We induced deletion of *Ntn1* at 6 weeks after birth (*Ntn1*^*ΔCAGGSCre/ΔCAGGSCre*^) and analysed mice 8 weeks later (Fig. 6a). *Ntn1* deletion caused an increase in the relative frequencies of myeloid cells, especially neutrophils in both peripheral blood and BM (Figure S3b–d). Strikingly, the frequency of HSCs in *Ntn1*^*ΔCAGGSCre/ΔCAGGSCre*^ BM was significantly reduced, while simultaneously the frequency of both MPP2 and MPP3/4 cells expanded (Fig. 6b, c and Figure S3e, f). In response to the induced *Ntn1* deletion, HSCs entered a more proliferative, less quiescent state, represented by an increase of HSCs in G2–S–M and a reduction in G0 (Fig. 6d). After *Ntn1* deletion, HSCs also expressed reduced levels of *Egr1*, while expression of *Cdk6*, as well as the differentiation associated genes *Gata1* and *Itga2b* increased (Fig. 6e). Finally, *Neo1* expression was upregulated in *Ntn1*^*ΔCAGGSCre/ΔCAGGSCre*^ HSCs, suggesting a compensatory upregulation in response to the absence of its ligand (Fig. 6e).

The observed reduced numbers of HSCs were even more pronounced at 5 months post *Ntn1* deletion, suggesting a progressive loss of HSCs after *Ntn1* deletion (Fig. 6f and Figure S3g, i). To test whether increased levels of NTN1 could alter HSC behaviour in vivo, we generated *CAGGS:Cre*^*ERT2*^*; LSL-Rosa26-Ntn1 mice* (*Ntn1-OE*) and induced Cre expression in 6-week-old animals, leading to a 30-fold increase of *Ntn1* levels in BM endothelial cells after 5 months (Figure S3h). While we found no difference in HSPC frequencies (Figure S3j), quiescent G0-HSCs increased, suggesting that *Ntn1* overexpression in the BM microenvironment leads to increased HSC quiescence in vivo (Fig. 6g and Figure S3k). In addition, the frequency of cycling HSCs 5 months after *Ntn1* deletion was significantly increased, reproducing the 2-month timepoint (Fig. 6d, g and Figure S3k). In summary, Ntn1 mediates HSC quiescence not only in culture but also in vivo and loss of *Ntn1* activates and progressively depletes quiescent, functional HSCs.

### Conditional *Ntn1* deletion impairs HSC function

To study, whether the Ntn1-mediated increase (*Ntn1-OE*) or reduction in HSC quiescence and frequency (*Ntn1* deletion) is associated with functional consequences, we competitively transplanted total BM of *Ntn1-OE, Ntn1*^*ΔCAGGSCre/ΔCAGGSCre*^ or control (*CAGGS-Cre*^*ERT2*^) mice 5 months after Tam induction (Fig. 6h). Upon *Ntn1-OE*, we neither observed any differences in peripheral blood leucocytes nor in HSC frequencies 4 months after transplantation (Fig. 6i, k). In contrast, *Ntn1* deletion led to a reduced contribution of CD45.2^+^ donor cells to peripheral blood leucocytes (Fig. 6i, j) accompanied with a strong reduction of HSC numbers 4 months after transplantation (Fig. 6k). Next, we addressed the engraftment potential of 200 purified HSCs (LSK, CD150^+^, CD48^−^, CD34^−^) isolated either from a microenvironment, in which Ntn1 was deleted for 5 months (*Ntn1*^*ΔCAGGSCre/ΔCAGGSCre*^) or expressed on normal levels (Fig. 6l). Two months post transplantation, the HSC frequency was significantly reduced compared to control HSC, which have developed in an Ntn1-proficient environment (Fig. 6m). These data show that HSCs derived from an *Ntn1*-deficient BM become functionally impaired and this self-renewal defect is not reversed by transplanting them back into an Ntn1-proficient recipient microenvironment.

### *Ntn1* expressed by arterioles maintains HSCs

**Fig. 7:**
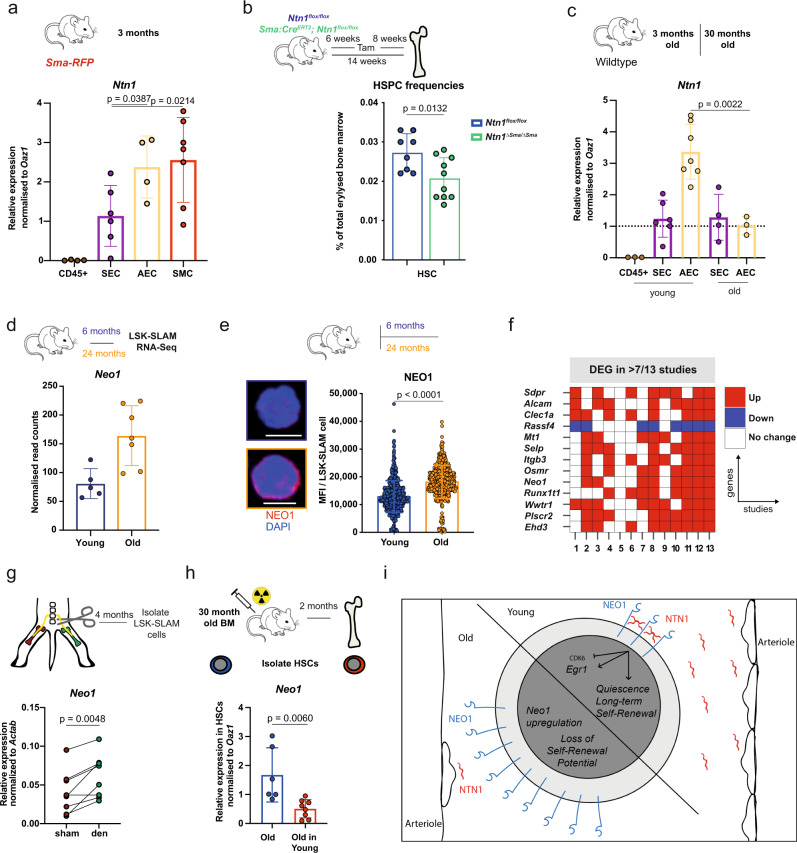
Loss of niche-derived Ntn1 induces Neo1 in HSC upon ageing. **a** Relative expression of *Ntn1* in CD45^+^ cells, SEC, AEC and RFP^+^ SMC derived from *Sma-RFP* mice; *n* = 4 (CD45/AEC), 6 (SEC) and 7 (SMA-RFP), two independent experiments. **b** Frequencies of HSCs in bone marrow of in *Ntn1*^*flox/flox*^ and *Ntn1*^*ΔSma/ΔSma*^ mice; *n* = 8 (flox) and 10 (∆SMA), three independent experiments. **c** Relative expression of *Ntn1* in SEC, AEC and CD45^+^ cells derived from young and old Wt mice; *n* = 3 (yCD45/oAEC), 4 (oSEC), 6 (ySEC) and 7 (yAEC), three independent experiments. **d** Normalized read counts of *Neo1* in young, and old LSK-SLAM cells; *n* = 5 (young) and 7 (old), FDR < 0.0001. **e** MFI of NEO1 in sorted 6 or 24 months LSK-SLAM cells; *n* = 592 (young)–593 (old). **f** Most abundant common DEGs in published ageing studies and own data, additional details in the “Methods” section. **g** Relative expression of *Neo1* in LSK-SLAM cells isolated from either denervated or healthy legs of individual mice; *n* = 8, two independent experiments. **h** Relative expression of *Neo1* in HSCs of aged mice, before and after 2 months post transplantation; *n* = 6 (before) and 8 (after), two independent experiments. **i** Model of Neo1/Ntn1 axis in young and old mice. For all panels, ±SD is shown. *n* indicates biological replicates. Scale bars in IF images are 5 μm. *P* value determined by two-tailed *t* test unless stated otherwise. Source data are provided as a Source Data file.

We next investigated which niche cells express *Ntn1*. By screening published datasets, we found that *Ntn1* is expressed at low levels in sinusoidal (SEC: CD45^−^, CD31^+^, Sca-1^medium^, Pdpn^+^) and at higher levels in arteriolar endothelial cells (AEC: CD45^−^, CD31^+^, Sca-1^high^, Pdpn^−^)^65^. In addition, *Ntn1* expression has been reported in periarteriolar smooth muscle cells (SMCs)^66^. To examine *Ntn1* expression within the BM niche, we isolated AECs, SECs, CD45^+^ hematopoietic and RFP^+^ cells derived from *Sma-RFP* (*smooth muscle actin-RFP*) reporter mice marking SMCs^67^ (Figure S4a, b). While we found no expression in CD45^+^ hematopoietic cells, we detected the highest *Ntn1* levels in AECs and SMCs (Fig. 7a). To investigate whether periarteriolar smooth muscle-derived Ntn1 regulates HSCs, we generated *Sma-Cre*^*ERT2*^*; Ntn1*^*flox/flox*^ mice, injected adult mice with Tam and studied HSCs 8 weeks after Cre induction. In line with depletion of HSCs upon global *Ntn1* deletion, we detected a decrease in HSCs in *Ntn1*^*ΔSmaCre/ΔSmaCre*^ animals compared to controls (Fig. 7b). This reduction was, however, not as strong as we observed upon global *Ntn1* deletion using CAGGS-Cre (Fig. 6), suggesting additional Ntn1 sources like AECs. As BM arterioles deteriorate upon ageing, leading to the loss of HSC maintaining SCF^23,24^, we isolated SECs and AECs from young and old Wt mice and found diminished *Ntn1* expression specifically in old AECs (Fig. 7c). When we investigated *Neo1* in aged HSCs, we found expression was still restricted to HSCs, but levels were significantly increased (Figure S4c), in line with our RNA-seq data from aged Wt chimeras (Figure S2d). To further confirm this, we performed RNA-seq of young and old LSK-SLAM cells. We found *membrane-associated* processes and *receptors* to be upregulated upon ageing (Figure S4d). Specifically, *Neo1* expression increased robustly on RNA and protein level in old HSCs (Fig. 7d, e). Several studies have previously compared transcriptional profiles of young vs. old HSCs (using different marker combinations). However, the studies showed a wide variety of DEGs with little consistency (Figure S4e). To identify consistently changed DEGs upon HSC ageing, we added 12 previously published transcriptome datasets of aged HSCs to our own study and performed a meta-analysis (Figure S4e). In these 13 datasets, not a single DEG was shared among ten or more studies, again highlighting the heterogeneity. Nevertheless, 13 genes were consistently differentially expressed in eight to nine datasets (Fig. 7f). Seven of these were receptors and one of these was *Neo1*, suggesting that Neo1 is one of the most consistently upregulated genes found upon HSC ageing.

It has recently been established that surgical BM denervation mirrors the phenotype of arteriolar degeneration upon ageing and thereby induces premature HSC ageing^24^. Therefore, we tested whether the observed *Neo1* upregulation during HSC ageing (Fig. 7d) or as a consequence of *Ntn1* deficiency (Fig. 6e) was recapitulated upon denervation-mediated induction of premature marrow ageing. One hind limb per Wt mouse was surgically denervated and LSK-SLAM cells 4 months after surgery were analysed. We found an increase in *Neo1* expression in HSCs of seven out of eight denervated femurs compared to sham-operated nerve-intact contralateral femurs of the same mice (Fig. 7g). The *Neo1* upregulation is consistent with a model that the normal or accelerated ageing process leads to a decrease in Ntn1 expression in the microenvironment, mediating a compensatory Neo1 upregulation to maintain signalling when its ligand Ntn1 becomes limiting.

Finally, we investigated whether the niche mediated upregulation of *Neo1* in HSCs of 30-month-old mice (NTN1^low^ environment) can be reversed by transplanting them into 2-month-old young mice (NTN1^high^ environment). Indeed, *Neo1* expression in HSCs significantly decreased again in young mice (Fig. 7h). These data further support the link between the level NTN1 production in the BM microenvironment and expression of its receptor *Neo1* on HSCs in young and old mice (Fig. 7i). However, the compensatory upregulation of *Neo1* expression due to age-dependent ligand deprivation is not sufficient to maintain NEO1 function, since ablation of either Ntn1 or Neo1 leads to proliferation and decreased self-renewal of HSCs, a hallmark of aged HSCs.

## Discussion

Here, we identify arteriolar niche-derived NTN1 ligand and its cognate HSC-specific receptor NEO1 as a novel ligand-receptor signalling axis regulating HSC quiescence and long-term self-renewal. This axis is deregulated upon ageing and loss of either of its components leads to functional HSC impairment. NTN1–NEO1 represents a novel intercellular and non-cell autonomous signalling network by which NTN1 produced by perivascular niches binds to HSCs to fine-tune HSC dynamics, in particular cell cycle activity and long-term self-renewal.

In agreement with Neo1 being specifically expressed by dHSCs (Fig. 1), *Neo1* is part of the *MoIO* signature marking functionally superior HSCs^50^. Expression of *Neo1* is also highest in Vwf^+^ HSCs residing on the top of the hematopoietic hierarchy^68^ and NEO1^+^ cells have recently been reported as a subpopulation within Hoxb5^+^ HSCs^42^. Intriguingly, *Dnmt3a* mutant HSCs show increased quiescence, as well as a robust upregulation of *Neo1* expression^69–71^, suggesting it as a potential target for *Dnmt3a* mutant hematopoietic disorders.

When characterizing *Neo1*-mutant hematopoiesis, we observed an initial increase in HSC numbers associated with loss of quiescence and subsequently loss of HSC self-renewal over time that correlated with decreased expression of *Egr1* and increased expression of *Cdk6*. Similarly, hematopoietic loss of *Egr1* leads to increased cycling and initial HSC expansion followed by a loss of engraftment potential upon serial transplantation^55^. Since we analysed a hypomorphic *Neo1* mouse model with severely decreased (>90%) but remaining minor expression^38,47,48^, our results possibly underestimate the biological relevance of Neo1 in HSCs. It has been reported, that ≈80% of *Neo1*^*gt/gt*^ mice die prenatally. The ones born develop hydrocephalus of varying degree, with around one in five displaying severe phenotypes with macroscopically visible “dome-shaped” skulls^48^. Since this was reproducible in our analysis, we used only *Neo1*^*gt/gt*^ mice without macroscopic features of hydrocephalus, which showed normal, healthy behaviour. In these *Neo1*^*gt/gt*^ mice, the HSC numbers were unchanged at the time of analysis. Nevertheless, we cannot formally exclude that additional factors such as neuronal stress may contribute to some extent to the described HSC phenotype in the primary Neo1^gt/gt^ mutants.

NEO1 can bind multiple neural guidance molecules, which mediate context-dependent effects. As an example, RGMs are known to inhibit neuronal migration^72^, while NTN1 acts as a chemoattractant for commissural axons^63^. In HSCs, we found NTN1, but neither RGMs nor BMP-2, to modulate HSC behaviour. This is intriguing because, in the developing bone, NEO1 modulates cartilage growth via canonical BMP signalling^73^. However, the relevance of BMP signalling for adult HSCs remains uncertain^74^.

Over the past years, the role of netrins in neurobiology, originally established using gene-trapped mice^62^, has been challenged by novel conditional *Ntn1* alleles^63,75,76^. When we repurposed these to investigate hematopoiesis, we found increased activation and progressive loss of HSC numbers as well as self-renewal potential after global deletion of *Ntn1*, mimicking the *Neo1*-mutant phenotype. Further, in vivo overexpression and in vitro stimulation with NTN1 enhanced HSC quiescence and increased engraftment potential of cultured HSCs upon transplantation, respectively. These results are in line with studies showing quiescence-inducing compounds that maintain HSC engraftment potential in vitro^11,29,77^ as well as studies that associate loss of self-renewal capability in vivo with divisional history^13,78,79^. Altogether, the data strongly suggest that NTN1 acts as a paracrine NEO1 ligand modulating HSC behaviour. Furthermore, Ntn1 has been described to support immature states of iPSCs and cancer stem cells^80,81^, suggesting that it maintains stemness in various settings. Here, we demonstrate that NTN1/NEO1 signalling increase NF-κB activation in HSCs, a pathway known to protect HSCs from exhaustion during stress, while the loss of p65 leads to hematopoietic failure^61,82^.

Within the BM niche, we found *Ntn1* to be expressed in AECs and SMC in line with the previous studies^65,83,84^. These as well as other perivascular cells secrete multiple molecules that support HSCs including SCF and CXCL12^26,27,65,83,85,86^. Upon ageing, BM arterioles are remodelled leading to a depletion of periarteriolar stromal cells and SCF, affecting hematopoiesis^23–25^. In line, NTN1 secretion by SMCs is known to guide axons of the sympathetic nervous system during arteriolar growth^66^. The connection between the sympathetic nervous system and arterioles is intriguing, as denervation disrupts BM arterioles and mediates accelerated HSC ageing^24^.

Our data strongly support the link between NTN1 production in the BM microenvironment and expression of its receptor Neo1 on HSCs. Loss of *Ntn1* expression in the niches during: (a) physiological ageing, (b) accelerated ageing by surgical denervation or (c) by genetic ablation results in compensatory upregulation of *Neo1* expression due to ligand deprivation, which, however, is not sufficient to maintain Neo1 function. Such a mechanism has also been observed for the Ntn1 receptors DCC and NEO1 upon loss of *Ntn1* during development^76^.

Collectively, our data suggest that NTN1 produced mainly by arteriolar niches preserves quiescence and self-renewal of HSCs via NEO1, while ageing-associated decline of Ntn1 leads to the gradual decrease of Neo1-mediated HSC self-renewal.

## Methods

### Contact for reagent and resource sharing

Further information and requests for resources and reagents should be directed to and will be fulfilled by the Lead Contact, Andreas Trumpp a.trumpp@dkfz.de. Certain materials are shared with research organizations for research and educational purposes only under an MTA to be discussed in good faith with the recipient.

### Experimental mouse models

#### *SCL-tTA; H2B-GFP* mice

This transgenic mouse line expresses the fusion protein histone H2B-GFP under the tetracycline-responsive regulatory element and the tTA-S2 transactivator from the endogenous *Scl* locus^10^. Doxycycline was supplemented in drinking water of 8–16-week-old mice for 150 as previously described^10^. To set the gates for GFP^+^ cells, age-matched *H2B-GFP* littermates were used. *SCL-tTA; H2B-GFP* mice were backcrossed to C57BL/6J.

C57BL/6J (CD45.2, CD45.1 or CD45.2/CD45.1) mice were either purchased from Envigo (the Netherlands) or Janvier Labs (France) or bred in-house.

#### *Gprc5c-GFP* mice (*Tg(Gprc5c-EGFP)JU90Gsat*)

This transgenic mouse line was previously generated by inserting an *EGFP* gene into a BAC clone at the initiating ATG codon of the first coding exon of the *Gprc5c* gene and this BAC clone was subsequently used to generate transgenic reporter mice^87^. Analysed mice were backcrossed to C57BL/6J.

#### *Myc-eGFP mice*

This transgenic mouse line expresses a fusion protein of c-Myc and eGFP^60^.

#### *FUCCI* mice *(B6-Tg(Gt(ROSA)26Sor-Fucci2)#Sia*)

This transgenic mouse line allows the identification of cell cycle phase via fluorescent fusion proteins, mice were sacrificed after 8–16 weeks^59^.

#### *Neo1*^*gt/gt*^ mice (*B6.129P2-Neo1*^*Gt(KST265)Byg/Mmmh*^)

These mice harbour a gene-trapped *Neo1* allele that leads to a strong reduction of *Neo1* expression^38^. For transplantation experiments, male and female animals 4–6 weeks of age were used. Control transplantations were always performed using gender-matched, wild-type littermates. For competitive transplantations, competitor BM was also age- and gender-matched.

#### *Ntn1*^*β-geo/+*^ mice (*Ntn1*^*Gt(ST629)Byg*^)

These mice harbour a gene-trapped *Ntn1* allele that leads to a strong reduction of *Ntn1*. Heterozygous mice can be used as reporter mice employing the β-gal reporter in the gene-trapped vector^62^.

#### *Ntn1*^*fl/fl*^ mice

This transgenic mouse line contains loxP sites flanking coding sequences containing both the principal ATG (based on *Ntn1* complementary DNA (cDNA) sequence NM_008744) and the cryptic ATG (based on *Ntn1* cDNA: BC141294) and the alternative promoter described in intron 3^63^. To generate global *Ntn1* deletion, we crossed *Ntn1*^*fl/fl*^ mice to *CAGGS-Cre*^*ERT2*^ mice (Jackson Laboratories). For smooth muscle-specific deletion, *Ntn1*^*fl/fl*^ mice were crossed to *Sma-Cre*^*ERT2*^ mice. For 8 weeks endpoints, *Ntn*^*fl/fl*^ crossings only female, and for 5 months endpoints, only male mice were analysed to reduce variability.

#### *+/LSL-Rosa26-Ntn1* mice

This transgenic mouse line was generated for this study. The human *NETRIN-1* was cloned in Rosa26-lox-stop-lox plasmid (Soriano). Mice were generated by SEAT CNRS Gustave Roussy Phenomin. We crossed these mice to *CAGGS:Cre*^*ERT2*^ mice (Jackson Laboratories), inducing global overexpression of Ntn1. To reduce variability, only male animals were analysed at 5 months after Cre induction.

#### *Sma-RFP mice* (*C.Cg-Tg(aSMA-RFP)#Rkl*

The mouse line harbours an RFP reporter for *Sma* and thereby allows identification of SMCs^67^. Sma-RFP mice are on a BALB/C background.

All other mouse models are on a B6J background.

All mice were bred in-house in the animal facilities of DKFZ, University Medical Center Groningen, INSERM or Albert-Einstein College of medicine under specific pathogen-free conditions in individually ventilated cages at 24°, a humidity of 80% with fixed day/night cycles of 12 h. According to German, French, American or Dutch guidelines, mice were euthanized by cervical dislocation and all animal procedures were performed according to protocols approved by the Regierungspräsidium Karlsruhe, Animal Care and Use Committee of Albert-Einstein College of Medicine, the Instantie voor Dierenwelzijn committee, Universitair Medisch Centrum Groningen/Rijksuniversiteit Groningen or University of Lyon local Animal Ethic Evaluation Committee. To reduce animal numbers, remaining BM/cDNA samples generated in this and previous studies were used whenever possible.

### Method details

#### pIC- or LPS-induced inflammatory stress

Mice were injected intraperitoneally with pIC (100 μg/mouse in 0.1 ml phosphate-buffered saline (PBS)), LPS (5 μg/mouse in 0.1 ml PBS) or PBS alone. Sixteen hours (LPS/pIC/PBS), 5 or 8 days (pIC/PBS) later, mice were sacrificed and BM cells were used for subsequent analysis.

#### Cell isolation and flow cytometry

Mouse BM cells were isolated, and HSCs and MPP1–4 progenitors defined by immune phenotype (lineage-negative (Lin^−^), Sca-1^+^, c-Kit^+^ (LSK), CD135^−/+^, CD150^−/+^, CD48^−/+^, CD34^−/+^, CD45.2 CD45.1/2, CD45.1) (see also Fig. 1a), or LSK-SLAM (Lin^−^ c-Kit^+^ CD150^+^ CD48^−^) purified by FACS and subsequently subjected to RNA-seq, population quantitative real-time PCR (qPCR) analysis, in vitro experiments, reconstitution assays or cytological analysis. Briefly, BM was isolated from pooled femora, tibiae, ilia and vertebrae by gentle crushing in PBS using a mortar and pistil. If no depletion of lineage-positive (Lin^+^) cells was performed, lysis of erythrocytes was performed using ACK Lysing Buffer (Thermo Fisher Scientific). To deplete Lin^+^ cells we used the Dynabeads Untouched Mouse CD4 Cells Kit (Invitrogen). Briefly, total BM was stained for 30 min with 100 μl/mouse of the Lineage Cocktail provided in the Dynabeads Untouched Mouse CD4 Cells Kit (Invitrogen) in PBS. Labelled cells were then incubated for 20 min with 1.5 ml/mouse of washed polyclonal sheep anti-rat IgG-coated Dynabeads provided in the Kit. Cells were depleted using a magnet, enriching for the Lin^−^ cell fraction. To purify HSC and MPP1–4, the Lin^−^ fraction was stained for 30 min using the following monoclonal antibodies: anti-lineage [anti-CD4 (clone GK1.5), anti-CD8a (53-6.7), anti-CD11b (M1/70), anti-B220 (RA3-6B2), anti-GR1 (RB6-8C5) and anti-TER119 (Ter-119)]; anti-CD117/c-Kit (2B8); anti-Ly6a/Sca-1 (D7); anti-CD34 (RAM34); anti-CD150 (TC15-12F12.2); anti-CD48 (HM48-1); anti-CD135 (A2F10); CD45.1 (A20); CD45.2 (104). The coupled fluorochromes used depended on the experiment to allow sorting of different fluorescent protein containing reporters or isolation of HSC from transplants. Monoclonal antibody conjugates were purchased from eBioscience, BD Bioscience or BioLegend. Cell sorting was then performed on a FACS Aria I, II and III, FACS Aria Fusion (Becton Dickinson) using BD FACSDiva v8.0.3 (BD Bioscience) or MoFlo Astrios or XDP cell sorters (Beckman Coulter). Sorted in Complete Stem Cell Medium (specified elsewhere) for in vitro culture, cytology and reconstitution experiments, or RNA lysis buffer (ARCTURUS PicoPure RNA Isolation Kit (Life Technologies, Invitrogen) for population RNA-seq or qPCR and stored at −80 °C.

For Figure S4c, long-term HSCs were characterized as Lin^−^Sca-1^+^c-Kit^+^CD48^−^CD150^+^, ST-HSCs as Lin^−^Sca-1^+^c-Kit^+^CD48^−^CD150^−^ and MPPs as Lin^−^Sca-1^+^c-Kit^+^CD48^+^CD150^−^. For isolation of committed progenitor subsets, the following markers were used for isolation: CLP (Lin^−^CD127^+^Sca-1^lo^c-Kit^lo^), CMP (Lin^−^CD127^−^Sca-1^−^c-Kit^−^CD34^+^CD16/CD32^hi^), GMP (Lin^−^CD127^−^Sca-1-c-Kit^+^CD16/CD32^hi^) and MEP (Lin^−^CD127^−^Sca-1^−^c-Kit^+^CD34^−^CD16/CD32^+^). Data were analysed using FlowJo, versions 6-10.5.3.

#### CDK6/NEO1 staining

For analyses of NEO1 expression, BM cells were isolated from mice and stained for HSC/MPP markers and sorted as described. Cells were plated on poly-l-lysine-coated slides and then fixed with BD Cytofix/Cytoperm Buffer (Beckton Dickinson). Subsequently, Cdk6 (Abcam) or Neo1 (Abcam) staining was performed in 0.1% Triton (Sigma) and 5% bovine serum albumin (BSA). Secondary antibodies coupled to AF-488 were used. After washing, slides were embedded in anti-fade reagent with 4′,6-diamidino-2-phenylindole (DAPI) (Invitrogen) and imaging was performed employing a Zeiss LSM 700 or LSM 710 confocal microscope using ZEN blue v2.5 (Zeiss international). Experimental replicates were always performed side by side and imaged in one session without change of laser intensities or gain to avoid technical bias and allow the comparability. DAPI signal was not used for quantification or normalization. The analysis was performed with FIJI v.2.0.

#### NEO1 staining (Fig. 7e)

A total of 4000–6000 long-term HSCs were seeded in spots of an immunofluorescent adhesion slide (VWR) and allowed to settle for 20 min. Cells were then fixed and permeabilized with Fixation and Permeabilization Solution (BD Biosciences) for 20 min on ice. Cells were then blocked with 4% BSA for 30 min at room temperature and then stained with 1:100 mouse Neo1 biotinylated antibody (R&D) at 4 °C. Cells were washed three times in 0.1% Triton X-100 PBS solution and stained with 1:500 secondary antibody streptavidin Alexa-647 for 1 h at 4 °C. After washing, coverslips were mounted in anti-fade reagent with DAPI (Invitrogen).

#### p65-GFP staining

After 48 h of the culture of 2000 HSCs/well, cells were plated on poly-l-lysine-coated slides and then fixed with BD Cytofix/Cytoperm Buffer (Beckton Dickinson). Subsequently, anti-GFP-488 (Abcam) staining was performed in 0.1% Triton (Sigma) 5% BSA for 1 h to increase the signal. After washing, coverslips were mounted in anti-fade reagent with DAPI (Invitrogen).

#### Cell cycle analysis

HSC/MPP surface staining (LSK, CD150, CD48, CD34) was performed on BM cells or in vitro treated HSPCs. Cells were fixed with BD Cytofix/Cytoperm Buffer (Beckton Dickinson). Subsequently, intracellular Ki-67 (BD Biosciences) staining was performed using PermWash solution (Beckton Dickinson). Prior to flow cytometry analysis, cells were stained with Hoechst 33342 (Invitrogen) or DAPI (Thermo Fisher).

#### Population RNA-seq

For Fig. 4e–j and Figure S2c–e, population RNA-seq data were generated as previously described^1^. Briefly, total RNA isolation was performed using ARCTURUS PicoPure RNA Isolation Kit (Life Technologies, Invitrogen) according to the manufacturer’s instructions. Total RNA was used for quality controls and for normalization of starting material. cDNA libraries were generated using 1 ng of total RNA for *Neo1*-deficient/Wt HSCs using the SMARTer Ultra Low RNA Kit for Illumina Sequencing (Clontech) according to the manufacturer’s indications. Sequencing was performed using the HiSeq2000 device (Illumina).

#### RNA-seq: young and old LSK-SLAM for Fig. 7d and Figure S4d

RNA was isolated from 15,000 LSK-SLAM cells (Lin^−^Sca-1^+^c-Kit^+^CD48^−^CD150^+^) using the Nucleospin XS Kit (Macherey Nagel) and quantified on Bionalyzer using RNA Pico 6000 Kit (Agilent). Ribosomal depletion was performed using a modified version of RiboZero Kit (Illumina). In all, 300 pg ribosomal-depleted RNA was used as input into TotalScript RNA-Seq Kit (Epicentre). Libraries were pooled and sequenced to 30–50 million reads on HiSeq 2500.

#### qPCR analysis

For qPCR, total RNA of 2000–10,000 cells were isolated as described above or using Nucleospin RNA XS Kit (Machery-Nagel) and reverse-transcribed using SuperScript VILO cDNA Synthesis Kit (Invitrogen) according to the manufacturer’s guidelines. For qPCR analysis, Fast SYBR Green Master Mix (Thermo Scientific) or LightCycler SYBR Green I (Roche) was used on a ViiA 7 Real-Time PCR System (Applied Biosystems) or a LightCycler 480 Instrument (Roche). RNA expression was normalized to *Oaz1*, *Act2b*, or *Hprt* housekeeping gene expression and presented as relative quantification (ratio = 2^−^^DDCT^). Primers were designed using the Universal ProbeLibrary Assay Design Center (Roche) or NCBI Primer-BLAST (NCBI) and ordered from Sigma. Primer sequences are available in the Supplementary Information file.

#### Reconstitution experiments

For the generation of full chimeras, 3 × 10^6^ total BM cells from 4-week-old Wt CD45.2 or *Neo1*^*gt/gt*^ CD45.2 mice were injected per recipient mouse.

For the generation of 50/50 chimeras, 1.5 × 10^6^ total BM cells from 4-week-old Wt CD45.2 or *Neo1*^*gt/gt*^ CD45.2 mice were mixed with 1.5 × 10^6^ total BM cells derived from CD45.1/2 mice so that a total of 3 × 10^6^ BM cells was injected per recipient mouse.

For transplantation after in vitro treatment, 500 sorted HSCs of CD45.1/2 or CD45.2 derived from 8- to 12-week-old animals were cultured for 48 h in respective conditions. Then, a well of the progeny of CD45.1/.2 was mixed to a well of the progeny of CD45.2 and then transplanted. A similar setup was used for the assessment of *Neo1* dependency of in vitro *Ntn1* treatment: 500 *Neo1*-deficient CD45.2^+^ HSCs were sorted after 8 months from old *Neo1* full chimeras and then incubated with or without Ntn1. These *Neo1-*deficient HSCs were mixed with 500 CD45.1/2 HSC incubated without Ntn1, derived from 8-week-old CD45.1/2 animals and then transplanted into individual recipients.

For secondary transplantations, 3 × 10^6^ total BM cells were isolated and transplanted.

For potency assessment of 100/500 CD45.2 Wt or *Neo1-*mutant HSCs were sorted from straight chimeras 15 months after transplantation, mixed with 1 × 10^5^ total BM cells from 8- to 12-week-old CD45.1/.2 mice and transplanted.

For assessment of HSC homing, 10 × 10^3^ LSK cells derived from CD45.2 Wt or *Neo1*-deficient mice were sorted and transplanted.

For the generation of 50/50 chimeras, 1.5 × 10^6^ total BM cells from 6.5-month-old Ntn1-OE/*Ntn1*^*ΔCAGGSCre/ΔCAGGSCre*^ or CAGGS-Cre mice, were mixed with 1.5 × 10^6^ total BM cells derived from CD45.1/2 mice so that a total of 3 × 10^6^ BM cells was injected per recipient mouse.

For transplantations of sorted HSC, 200 HSCs from 6.5-month-old *Ntn1*^*ΔCAGGSCre/ΔCAGGSCre*^ or CAGGS-Cre were mixed with 1 × 10^6^ total spleen cells from CD45.1/2 mice and injected into the recipient.

For transplantation of a 30-month-old HSC, 3 × 10^6^ total BM cells were isolated and injected into recipients.

For all experiments, cells were transplanted into fully irradiated (2 × 5 Gy) B6J mice (CD45.1). Contribution of CD45.2 or CD45.1/.2 donor cells was monitored in peripheral blood approximately every 4 weeks post transplantation in all transplantations using either LSRII, LSR Fortessa.

Outcome was addressed by absolute blood counts or flow cytometry using the following monoclonal antibodies: anti-CD45.1 (A20); anti-CD45.2 (104); anti-CD4 (clone GK1.5), anti-CD8a (53-6.7), anti-CD11b (M1/70), anti-B220 (RA3-6B2), anti-GR1 (RB6-8C5). The applied fluorochromes depended on the experiment. For endpoint analysis of chimera animals, BM stainings were performed as following: anti-lineage [anti-CD4 (clone GK1.5), anti-CD8a (53-6.7), anti-CD11b (M1/70), anti-B220 (RA3-6B2), anti-GR1 (RB6-8C5) and anti-TER119 (Ter-119)]; anti-CD117/c-Kit (2B8); anti-Ly6a/Sca-1 (D7; anti-CD34 (RAM34); anti-CD150 (TC15-12F12.2−; anti-CD48 (HM48-1); anti-CD45.1 (A20); anti-CD45.2 (104)). Monoclonal antibody conjugates were purchased from eBioscience, BD Bioscience or BioLegend.

#### Tam induction schema

For *Sma*:*Cre*^*ERT2*^*; Ntn1*^*fl/fl*^*, CAGGS:Cre*^*ERT2*^*; Ntn1*^*fl/fl*^ *Ntn1*^*fl/fl*^ and *CAGGS:Cre*^*ERT2*^*; LSL-Rosa26-Ntn1 mice* 3 Tam (Sigma-Aldrich) injections in 1 week were performed 4–6-week-old animals. At 8 weeks or 5 months after Cre induction mice were sacrificed and analysed.

#### BrdU analysis

Full chimeras of *Neo1*-mutant or Wt CD45.2 BM were injected intraperitoneally with 0.2 ml BrdU (1.8 mg/ml; Sigma) 4 months after transplantation and sacrificed 48 h after injection. Then, HSC surface staining was performed as described above, cells were processed and then staining with anti-BrdU (BD Bioscience) antibody (1:30) was performed.

#### HSPCs plating and in vitro treatment

In all, 5000 LSK-SLAM cells for RNA analysis, or 5000–10,000 HSCs for imaging or cell cycle analysis were sorted into and then cultured in Complete Stem Cell Medium (StemPro-34 SFM, Life Technologies containing 50 ng/ml SCF and 25 ng/ml TPO (all Preprotech), 100 U/ml penicillin/streptomycin, 2 mM l-glutamine, StemPro-34 supplement as recommended). Cells were cultured in 96-well ultralow attachment plates (Corning) and were treated with either recombinant 1 μl/ml Ntn1 (R&D), 1 μl/ml Rgm-a (R&D), 1 μl/ml Rgm-b (R&D), or 200 ng/ml Bmp-2 (R&D) in addition to the standard cytokines. The concentration of JSH-23 was 6 μM. At 48 h after plating, cells were used for downstream procedures such as RNA isolation, imaging or flow cytometry as described in its respective chapter.

#### BM denervation by the transaction of femoral and sciatic nerves

Denervation of the femoral and tibial BM was done as previously described^24^. The femoral nerve was localized after its exit from the vertebral column deep in the psoas muscle. This was accomplished with a midline abdominal incision; the intestines were gently moved aside to visualize the psoas muscle. An incision was made in the psoas to visualize the femoral nerve, and a 1 cm section of the nerve was excised. Deep to the psoas (through the incision), the sciatic nerve was visualized in close proximity to the iliac crest of the pelvis, and a 1 cm section of the nerve was excised. For sham operation, both femoral and sciatic nerves were exposed by surgery, but were left intact.

#### BM digestion for stromal cell isolation

For isolation of stromal cells, we thoroughly cleaned dissected bones, crushed and digested them for 1 h in RPMI with 2% fetal calf serum as well as 0.25% collagenase Type I (Gibco). After digestion was stopped with 10% fetal calf serum-containing medium, red cell lyses, lineage depletion, staining and cell sorting were performed as described above.

### Quantification, statistics and reproducibility

#### Standard quantifications, display and experimental design

Statistical analysis was performed with two-tailed paired Student’s *t* test or two-way analysis of variance using Fisher’s least significant difference for multiple comparisons as indicated in the respective figure legend. All data are presented as mean ± SD. For box and whiskers plots, error bars depict min to max values, the box is defined at 25–75th percentile and the median is marked with an additional line. Please see figure legends for detailed information. GraphPad Prism 7/8 was used for statistical analysis. The number of independent experiments is indicated in the respective figure legends. Sample exclusion was only done as a result of premature mouse death, infection or clear mistakes in sample processing.

#### RNA-seq analysis

For RNA-seq of *Neo1-mutant* and control HSCs, the following pipeline was used: sequenced read fragments were mapped to the mouse reference genome GRCm38 using STAR (STAR_2.6.1a)^88^. Expression counts estimates were generated using HTSeq (htseq-0.9.1)^89^. DESeq2 (DESeq2_1.20.0)^90^ was used to test for differential expression; results were considered significant at a *p*.adj. value <0.05. The analysis was performed in R-studio v3.5.2 (www.r-project.org).

For Fig. 7d, f and Figure S4d, differential expression was calculated using egdeR (3.24.3).

#### Downstream analysis

GSEA was performed using the GSEA software (3.0) on pre-ranked differential expression lists.

### Reporting summary

Further information on research design is available in the Nature Research Reporting Summary linked to this article.

## Supplementary information

Supplementary Information

Reporting Summary

Description of Additional Supplementary Files

Supplementary Data 1

Supplementary Data 2

Supplementary Data 3

## Data Availability

RNA-seq data have been deposited in online repositories: data linked to Fig. 4: MTAB-7902, data linked to Fig. 7: GSE128050. Expression data from young and old *Neo1-*mutant or control HSC can be found in Supplementary Data 1. Expression data from the analysis of young and old LSK-SLAM cells can be found in Supplementary Data 2. Source data for all seven figures and four Supplementary Figures are available in Supplementary Data 3. Nucleotide sequences and additional source data are available upon reasonable request to the corresponding author.
