# Supplementary material for: Niche derived netrin-1 regulates hematopoietic stem cell dormancy via its receptor neogenin-1
Source: Nat Commun. 2021 Jan 27;12:608. doi: 10.1038/s41467-020-20801-0 (PMC7840807; doi:10.1038/s41467-020-20801-0)
Supplement: Supplementary file 1 — Supplementary Information [file 41467_2020_20801_MOESM1_ESM.pdf]

Niche Derived Netrin-1 Regulates Hematopoietic Stem Cell Dormancy via its Receptor Neogenin-1

Renders et al.

Supplemental Figure 1

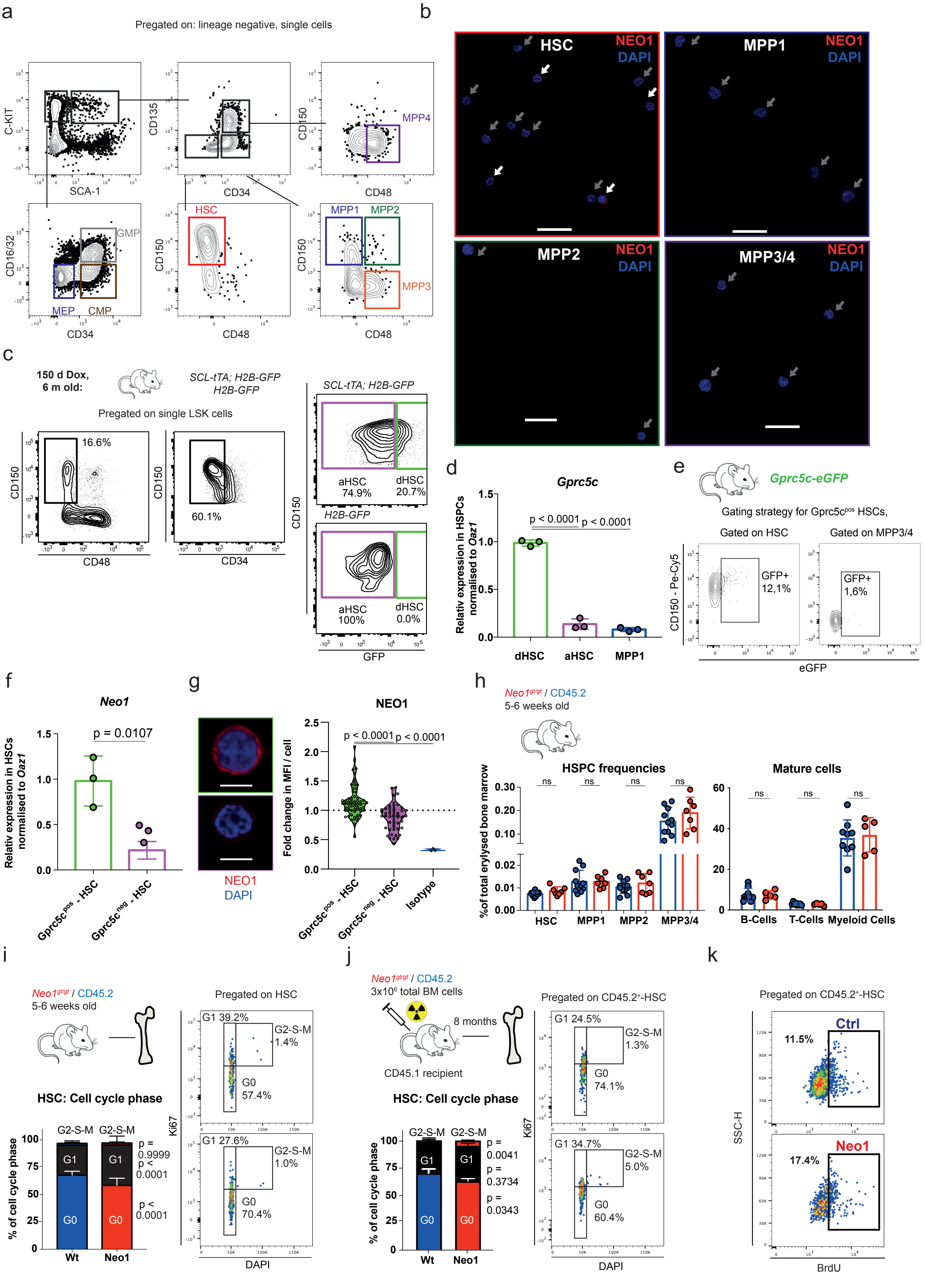

Supplemental Figure 1: Additional data regarding Neo1 expression in HSPC and Neo1-mutant mice

a) Gating strategy for HSPC as sorted in Figure 1b/c.

b) Representative images as depicted for Figure 1c. White arrows indicate NEO1 high, gray arrows NEO1 low cells. Scalebar is 20 µm

c) Gating strategy for label retaining cells (LRC) in *SCL-tTA; H2B-GFP* as well as *H2B*-GFP control mice chased for 5 months with Doxycyclin.

d) Relative expression of *Gprc5c* in HSPCs from *SCL-tTA; H2B-GFP*, after 5 months chase; n = 3.

e) Gating strategy for Gprc5c-GFP positive HSCs.

f) Relative expression of *Neo1* in HSPCs from *Gprc5c-GFP* mice; n = 3.

g) Fold change of MFI / cell of NEO1 in $Gprc5c^{+}$- and $Gprc5c^{-}$ -HSCs from *Gprc5c-GFP* mice; n = 3 (Isotype), 51 ($Gprc5c^{-}$), 61 ($Gprc5c^{+}$), 2 independent experiments.

h) Absolute frequencies of HSPCs derived from 5-6 weeks old Wt and $\textit{Neo1}^{gt/gt}$ bone marrow; n = 7(Neo1)-11(Ctrl), 5 independent experiments and of mature bone marrow cells; n=5(Neo1)-9(Ctrl), 4 independent experiments.

i) Representative FACS plots and analysis of HSCs cell cycle phases derived from 5-6 weeks old Wt and $\textit{Neo1}^{gt/gt}$ mice; n = 7(Neo1)-11(Ctrl); 5 independent experiments.

j) Representative FACS plots and analysis of cell cycle phases of $CD45.2^{+}$ HSCs derived full Wt and $\textit{Neo1}^{gt/gt}$ chimeras 8 months after transplantation; n = 8(Ctrl)-10(Neo1), 2 independent experiments.

k) Representative FACS plots of BRDU in $CD45.2^{+}$ HSCs in full Wt and $\textit{Neo1}^{gt/gt}$ chimeras at 4 months after transplantation.

For all panels, ± SD is shown. n indicates biological replicates. Scale bars in IF images are 5 µm unless stated otherwise. P-value determined by two-tailed t test unless stated otherwise. Source data are provided as a Source Data file.

Supplemental Figure 2

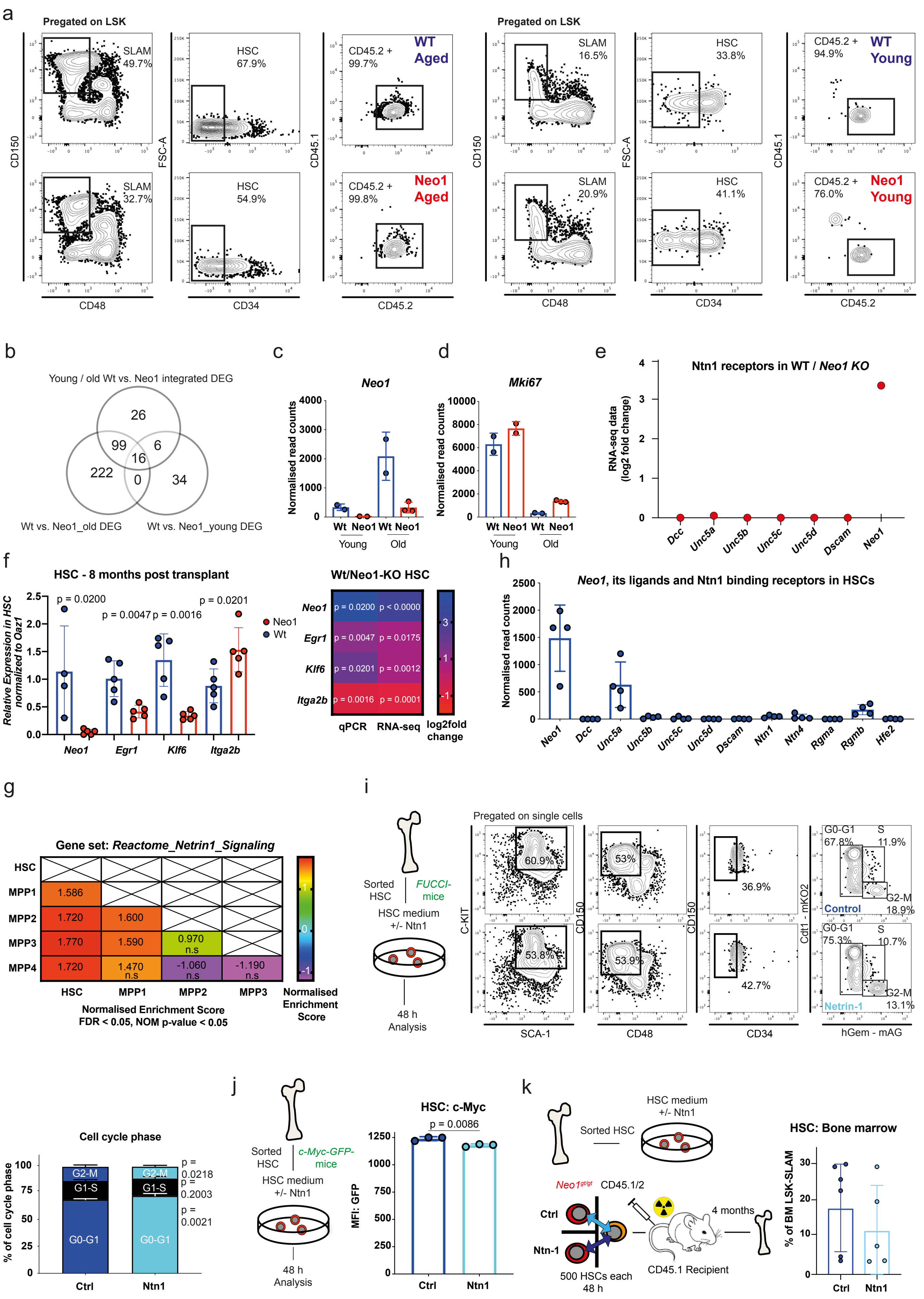

Supplemental Figure 2: Additional data regarding Neo1 RNA-seq, Ntn1 signalling in HSPC and Ntn1 in vitro experiments

a) Representative FACS plots for gating strategy of HSCs in full Wt and *Neo1*$^{gt/gt}$ chimeras at 4 and 15 months after transplantation as used for figure 4.

b) Venn Diagram indicating the overlap of DEG with an adjusted p-value < 0.05 from RNA-seq of "young" and "old" Wt *vs. Neo1*$^{gt/gt}$ HSCs as well as integrated young and old Wt vs *Neo1*$^{gt/gt}$

c) Normalised read counts of from *Neo1* in HSCs from young and old Wt and *Neo1*$^{gt/gt}$ chimeras, n= 4 (Ctrl)-5 (Neo1).

d) Normalised read counts of from *Mki67* in HSCs from young and old Wt and *Neo1*$^{gt/gt}$ chimeras, n= 4 (Ctrl)-5 (Neo1).

e) Fold change of Ntn1 receptors in Wt *vs. Neo1*$^{gt/gt}$ HSCs.

f) Relative expression of *Neo1*, *Egr1*, *Klf6* and *Itga2b* in HSCs derived from Wt and *Neo1*$^{gt/gt}$ chimeras eight month after transplantation and heatmap comparing qRT-PCR changes with RNA-seq changes displayed are log2fold changes. n=4 (Wt)-6(*Neo1*$^{gt/gt}$) .

g) Normalised read counts of Neo1 ligands and Ntn1 receptors in HSCs, derived from Cabezas-Wallscheid et al.

h) GSEA of a Ntn1 Gene set in all HSPC comparisons, expression changes derived from Cabezas-Wallscheid et al., FDR < 0.05, NOM p-value < 0.05.

i) Workflow, quantification and representative cell cycle plots of HSCs addressed with *FUCCI-mice* 48 h after Ntn1 treatment; n = 7.

j) Workflow and quantification of GFP levels in HSCs derived from *c-Myc-GFP* mice, 48 h after Ntn1 treatment; n = 3.

k) Workflow and chimerism of BM LSK-SLAM cells 4 months after competitive transplantation of Control- *vs.* Ntn1 treated *Neo1*$^{gt/gt}$ HSCs; n = 5 (Ntn1)-6(Ctrl).

For all panels, ± SD is shown. n indicates biological replicates. P-value determined by two-tailed t test unless stated otherwise. Source data are provided as a Source Data file.

Supplemental Figure 3

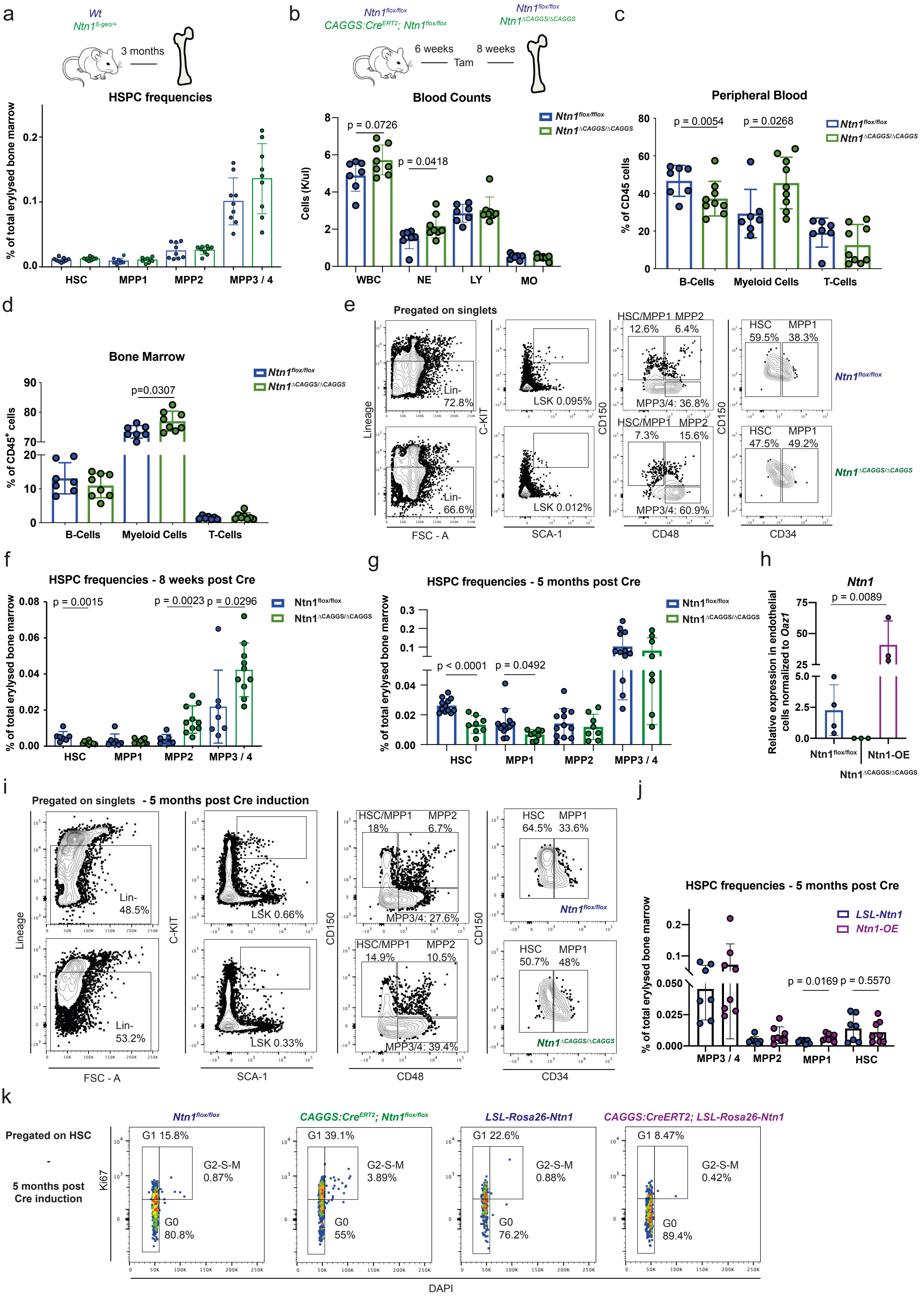

Supplemental Figure 3: Additional data regarding Ntn1 transgene animals

a) Frequencies of HSPCs in *Ntn*$^{ß\text{-}geo/+}$ or Wt mice; n = 9, 2 independent experiments.

b) Absolute blood counts derived from *Ntn1*$^{flox/flox}$ and *Ntn1*$^{\Delta CAGGS/\Delta CAGGS}$ mice, 8 weeks after deletion; n = 7(flox)-8(ΔCAGGS), 2 independent experiments.

c) Frequencies of differentiated cells in peripheral blood of *Ntn1*$^{flox/flox}$ and *Ntn1*$^{\Delta CAGGS/\Delta CAGGS}$ mice, 8 weeks after deletion; n = 7(flox)-8(ΔCAGGS), 2 independent experiments.

d) Frequencies of differentiated cells in bone marrow of *Ntn1*$^{flox/flox}$ and *Ntn1*$^{\Delta CAGGS/\Delta CAGGS}$ mice, 8 weeks after deletion; n = 7(flox)-8(ΔCAGGS), 2 independent experiments.

e) Gating strategy for analysis of HSPC frequencies in *Ntn1*$^{flox/flox}$ and *Ntn1*$^{\Delta CAGGS/\Delta CAGGS}$ mice, 8 weeks after deletion.

f) Frequencies of BM HSPCs in *Ntn1*$^{flox/flox}$ and *Ntn1*$^{\Delta CAGGS/\Delta CAGGS}$ mice, 8 weeks after deletion; n = 7(flox)-10(ΔCAGGS), 2 independent experiments.

g) Frequencies of BM HSPCs in *Ntn1*$^{flox/flox}$ and *Ntn1*$^{\Delta CAGGS/\Delta CAGGS}$ mice, 5 months after deletion; n = 8(ΔCAGGS)-12(flox)-, 3 independent experiments.

h) Relative expression of *Ntn1* in endothelial cells derived from *Ntn1*$^{flox/flox}$, *Ntn1*$^{\Delta CAGGS/\Delta CAGGS}$ *or Ntn1-OE mice*, 5 months after deletion; n = 3(ΔCAGGS/OE)-4(flox).

i) Gating strategy for analysis of HSPC frequencies in *Ntn1*$^{flox/flox}$ and *Ntn1*$^{\Delta CAGGS/\Delta CAGGS}$ mice, 8 weeks after deletion.

j) Frequencies of BM HSPCs in LSL-Rosa26-*Ntn1* and *Ntn1-OE* mice, 5 months after deletion; n = 7(LSL)-8(OE), 3 independent experiments.

k) Gating strategy for cell cycle analysis as depicted in Figure 6g

For all panels, ± SD is shown. n indicates biological replicates. P-value determined by two-tailed t test unless stated otherwise. Source data are provided as a Source Data file.

Supplemental Figure 4

a

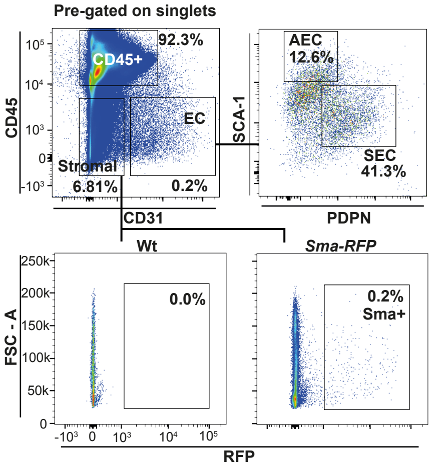

b

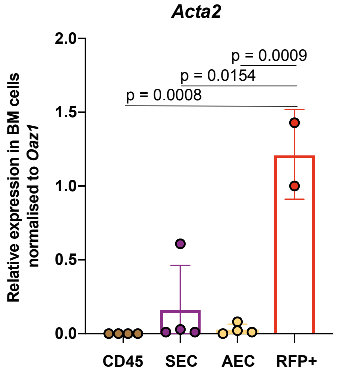

c

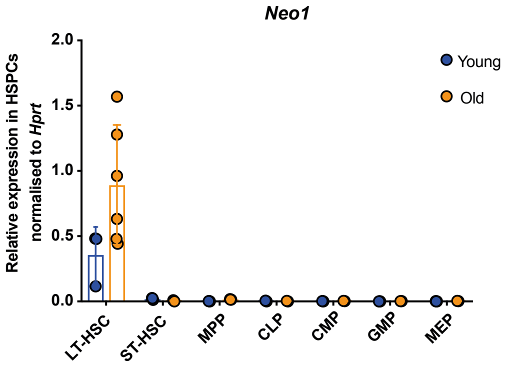

LT-HSC: Lin$^-$, SCA-1$^+$, c-KIT$^+$, CD48$^-$, CD150$^+$
ST-HSC: Lin$^-$, SCA-1$^+$, c-KIT$^+$, CD48$^-$, CD150$^-$
MPP: Lin$^-$, SSCA-1$^+$, c-KIT$^+$, CD48$^+$, CD150$^-$
CLP: Lin$^-$, CD127$^-$, SCA-1^{lo}, C-KIT^{lo}
CMP: Lin$^-$, CD127$^-$, SCA-1$^-$, C-KIT$^-$, CD34$^+$, CD16/ CD32^{hi}
GMP: Lin$^-$, CD127$^-$, SCA-1$^-$, C-KIT$^+$, CD16/ CD32^{hi}
MEP: Lin$^-$, CD127$^-$, SCA-1$^-$, C-KIT$^+$, CD34$^-$, CD16/ CD32$^+$

d

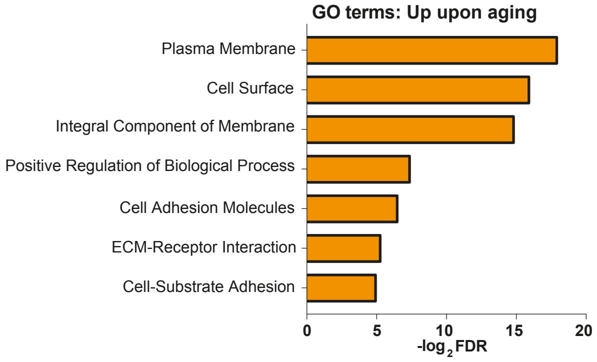

e

Study	Immunophenotype	DEGs	Source	ID
Beerman *et al.* 2013	LSK CD48$^-$ CD150$^+$	457	manuscript	1
Bersenev *et al.* 2012	LSK CD48$^-$ CD150$^+$	372	GEO	2
Chambers *et al.* 2007	LSK	2928	GEO	3
Flach *et al.* 2014	LSK CD48$^-$ CD150$^+$	630	GEO	4
Kischner *et al.* 2017	CD45$^+$ LSK CD48$^-$ CD150$^+$ EPCR$^+$	150	manuscript	5
Kowalcyk *et al.* 2015	LSK CD48$^-$ CD150$^+$	77	manuscript	6
Noda *et al.* 2009	LSK CD34$^-$	169	manuscript	7
Norddahl *et al.* 2011	LSK FLK2$^-$ CD34$^-$ CD150$^+$	832	GEO	8
Quere *et al.* 2014	LSK	204	manuscript	9
Rossi *et al.* 2005	LSK FLK2$^-$ CD34$^-$	978	manuscript	10
Own data	LSK CD48$^-$ CD150$^+$	180	manuscript	11
Sun *et al.* 2014	SP$^-$ LSK CD48$^-$ CD150$^+$	2634	manuscript	12
Wahlestedt *et al.* 2013	LSK CD48$^-$ CD150$^+$	490	manuscript	13

f

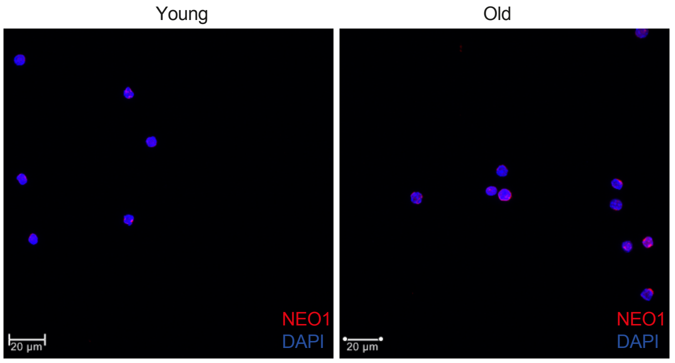

Supplemental Figure 4: Adittional data regarding Neo1/ Ntn1 expression in aged mice

a) Gating strategy for AEC, SEC and SMC in *Sma-RFP* mice.

b) Relative expression of *Acta2* in bone marrow cells; n = 2(RFP+)-4(SEC/AEC/CD45).

c) Relative expression of *Neo1* in HSPCs of 6- or 24 month-old mice. Mice were housed in ERIBA, Groningen; n = 3 (young),2-6(old).

d) G0-term analysis on DEGs upregulated upon ageing in RNA-seq of LSK-SLAM cells derived from 6- and 24 month-old mice.

e) Overview of ageing datasets used for Figure 1D, the respective immunophenotype, DEGs detected, ID in Figure 7f and reference is indicated.

f) Representative images as analysed in Figure 7e.

For all panels, ± SD is shown. n indicates biological replicates. Scale bars in IF images are 20 µm. P-value determined by two-tailed t test unless stated otherwise. Source data are provided as a Source Data file.

Supplemental Methods

Primers used for quantitative RT-PCR in the study:

Gene	Forward-Primer	Reverse-Primer
Acta2	CTC TCT TCC AGC CAT CTT TCA T	TAT AGG TGG TTT CGT GGA TGC
Cdk6	TGT GCA CAC ATC AAA CAA CCT	AGG TTG TTT GAT GTG TGC ACA
Gprc5c	GAG ATG GCC CTG ATG CAC	CAG GGT TGA GTT GGC ACT G
Itga2b	CAA CCG AGA CGG CTA TAA TGA	CAC TTG GCC CTG ACC ACT
Oaz1	TTT CAG CTA GCA TCC TGT ACT CC	GAC CCT GGT CTT GTC GTT AGA
Sdha	AAG TTG AGA TTT GCC GAT GG	CAG AAG TCG ATG CAG AAC CA
Egr1	AGT TCC AAC GCC CTC TGA C	ACA GGA ACC CGA ACC ACA
Hprt	GAC TTG CTC GAG ATG TCA	TGT AAT CCA GCA GGT CAG
Gata1	TCC CAG TCC TTT CTT CTC TCC	TGA GGC CAG AGA GTG TGT GA
Cd48	GTG TCT GGT CCT GGA ACT GC	GAT ATA AAT GCC ACC ACC GG
cMyc	CAC CAG CAG CGA CTC TGA	GGG GTT TGC CTC TTC TCC
Actb	GCT TCT TTG CAA GCT CCT TCG T	ATC GTC ATC CAT GGC GAA CT
Ntn1	GCA AGC TGA AGA TGA ACA TGA	CTT TGT CGG CCT TCA GGA
Neo1	GCA TAA CCT CGG ACC ACA AT	GCT GCT CTC ACA GTC AAT GG

Datasets used for Expression analysis in young vs. old HSPC:

Study	PMID	URL
Bersenev 2012	22812478	https://www.ncbi.nlm.nih.gov/geo/query/acc.cgi?acc=GSE39553
Chambers 2007	17676974	https://www.ncbi.nlm.nih.gov/geo/query/acc.cgi?acc=GSE6503
Flach 2014	25079315	https://www.ncbi.nlm.nih.gov/geo/query/acc.cgi?acc=GSE48893
Grover 2016	27009448	https://www.ncbi.nlm.nih.gov/geo/query/acc.cgi?acc=GSE70657
Kirshner 2017	28538171	https://www.ncbi.nlm.nih.gov/geo/query/acc.cgi?acc=GSE87631
Kowalczyk 2015	26430063	https://www.ncbi.nlm.nih.gov/geo/query/acc.cgi?acc=GSE59114
Mann 2018	30540934	https://www.ncbi.nlm.nih.gov/geo/query/acc.cgi?acc=GSE100428
Norddahl 2011	21549326	https://www.ncbi.nlm.nih.gov/geo/query/acc.cgi?acc=GSE27686
Sun 2014	24792119	https://www.ncbi.nlm.nih.gov/geo/query/acc.cgi?acc=GSE47819
Wahlestedt 2013	23476050	https://www.ncbi.nlm.nih.gov/geo/query/acc.cgi?acc=GSE44923
Maryanovich 2018	29736022	https://www.ncbi.nlm.nih.gov/geo/query/acc.cgi?acc=GSE109546

Antibodies used for flow cytometry or stainings

For flow cytometry antibodies, multiple different lots have been used over the course of this study.

anti-mouse CD4-PE-Cy7 (clone: GK1.5) Use: 1:1000	eBioscience	Cat#25-0041-82; RRID: AB_469576
anti-mouse CD8a-PE-Cy7 (clone: 53-6.7) Use: 1:1000	eBioscience	Cat#25-0081-81; RRID: AB_469583
anti-mouse CD11b-PE-Cy7 (clone: M1/70) Use: 1:1000	eBioscience	Cat#25-0112-82; RRID: AB_469588
anti-mouse B220-PE-Cy7 (clone: RA3-6B2) Use: 1:1000	eBioscience	Cat#25-0452-81; RRID: AB_469626
anti-mouse Ter119-PE-Cy7 (clone: TER-119) Use: 1:1000	eBioscience	Cat#25-5921-81; RRID: AB_469660
anti-mouse Gr1-PE-Cy7 (clone: RB6-8C5) Use: 1:1000	eBioscience	Cat#25-5931-82; RRID: AB_469663
anti-mouse CD117(c-Kit)-APC (clone: 2B8) Use: 1:1000	eBioscience	Cat#17-1171-82; RRID: AB_469430
anti-mouse Ly-6A/E(Sca-1)-APC-Cy7 (clone: D7) Use: 1:1000	BD Biosciences	Cat#560654; RRID: AB_1727552
anti-mouse CD150-PE-Cy5 (clone: TC15) Use: 1:1000	BioLegend	Cat#115912; RRID: AB_493598
anti-mouse CD48-PB (clone: HM48-1) Use: 1:1000	BioLegend	Cat#103418; RRID: AB_756140
anti-mouse CD135-PE (clone: A2F10) Use: 1:1000	eBioscience	Cat#12-1351-81; RRID: AB_465858
anti-mouse CD34-FITC (clone: RAM34) Use: 1:50	eBioscience	Cat# 11-0341-82; RRID:AB_465021
anti-human Ki67-AF647 (clone: B56) Use: 1:30	BD Biosciences	Cat#558615; RRID: AB_647130
BD APC BrdU Flow Kit	BD Biosciences	Cat#552598
anti-mouse CD45-FITC (clone: 30-F11) Use: 1:500	eBioscience	Cat # 11-0451-82, RRID:AB_465050
anti-mouse CD45.1-PB (clone: A20) Use: 1:500	eBioscience	Cat#25-0453-82; RRID: AB_469629
anti-mouse CD45.2-FITC (clone: 104) Use: 1:500	eBioscience	Cat #11-0454-82; RRID:AB_465061
Anti-mouse CD127- PE (clone: SB/199) Use: 1:500	BioLegend	Cat#121111; , RRID:AB_493510
Anti-mouse CD16/32- PE-cy7 (clone: 93) Use: 1:100	BioLegend	Cat#101317, RRID:AB_2104157
Anti-GFP AF488 Use: 1:500	abcam	Cat#ab192863, GR201295-1
anti-mouse Cdk6 (clone:K6.83) Use: 1:500	abcam	Cat#ab77674, RRID:AB_1566039, Lot: 268274-4
anti-mouse Neo1(clone: RM0124-3G55) Use: 1:500	Abcam	Cat#ab86577; RRID:AB_1925240, Lot: GR137-230-1
anti-mouse Neo1-Biotin Use: 1:500	R&D	Cat#BAF1079; RRID:AB_2251295
Anti-mouse Pdpn- APC (clone: 8.1.1) Use: 1:100	Biolegend	Cat# 127409, RRID:AB_10612940
Anti-mouse CD31- BV421 (clone: 390) Use: 1:100	Biolegend	Cat# 102424, RRID:AB_2650892
