# Supplementary material for: Niche derived netrin-1 regulates hematopoietic stem cell dormancy via its receptor neogenin-1
Source: Nat Commun. 2021 Jan 27;12:608. doi: 10.1038/s41467-020-20801-0 (PMC7840807; doi:10.1038/s41467-020-20801-0)
Supplement: Supplementary file 2 — Reporting Summary [file 41467_2020_20801_MOESM2_ESM.pdf]

nature research

Corresponding author(s): Nina Cabezas-Wallscheid
Andreas Trumpp

Last updated by author(s): 22/10/2020

# Reporting Summary

Nature Research wishes to improve the reproducibility of the work that we publish. This form provides structure for consistency and transparency in reporting. For further information on Nature Research policies, see Authors & Referees and the Editorial Policy Checklist .

## Statistics

For all statistical analyses, confirm that the following items are present in the figure legend, table legend, main text, or Methods section.

| n/a | Confirmed | |
|---|---|---|
| ☐ | ☒ | The exact sample size (*n*) for each experimental group/condition, given as a discrete number and unit of measurement |
| ☐ | ☒ | A statement on whether measurements were taken from distinct samples or whether the same sample was measured repeatedly |
| ☐ | ☒ | The statistical test(s) used AND whether they are one- or two-sided<br>*Only common tests should be described solely by name; describe more complex techniques in the Methods section.* |
| ☐ | ☒ | A description of all covariates tested |
| ☐ | ☒ | A description of any assumptions or corrections, such as tests of normality and adjustment for multiple comparisons |
| ☐ | ☒ | A full description of the statistical parameters including central tendency (e.g. means) or other basic estimates (e.g. regression coefficient) AND variation (e.g. standard deviation) or associated estimates of uncertainty (e.g. confidence intervals) |
| ☐ | ☒ | For null hypothesis testing, the test statistic (e.g. *F*, *t*, *r*) with confidence intervals, effect sizes, degrees of freedom and *P* value noted<br>*Give P values as exact values whenever suitable.* |
| ☒ | ☐ | For Bayesian analysis, information on the choice of priors and Markov chain Monte Carlo settings |
| ☒ | ☐ | For hierarchical and complex designs, identification of the appropriate level for tests and full reporting of outcomes |
| ☒ | ☐ | Estimates of effect sizes (e.g. Cohen's *d*, Pearson's *r*), indicating how they were calculated |

*Our web collection on statistics for biologists contains articles on many of the points above.*

## Software and code

Policy information about availability of computer code

| | |
|---|---|
| Data collection | BD FACSDiva v8.0.3 (Flowcytometers and sorters, BD Bioscience), ZEN blue v2.5 (Zeiss international) |
| Data analysis | FlowJo, Versions 6-10.5.3, statistical analysis with Graphpad Prism, versions 6-8.1.2, R-studio v3.5.2 (www.r-project.org), GSEA software v3.0 (Broad institute), Bioconductor scripts: STAR_2.6.1a, HTSeq_0.9.1, ggplot2_3.1.0, DESeq2_1.20.0, edgeR_3.24.3, FIJI v.2.0 |

For manuscripts utilizing custom algorithms or software that are central to the research but not yet described in published literature, software must be made available to editors/reviewers. We strongly encourage code deposition in a community repository (e.g. GitHub). See the Nature Research guidelines for submitting code & software for further information.

## Data

Policy information about availability of data

All manuscripts must include a data availability statement. This statement should provide the following information, where applicable:

- Accession codes, unique identifiers, or web links for publicly available datasets
- A list of figures that have associated raw data
- A description of any restrictions on data availability

RNA-seq data has been deposited in online repositories: Data linked to figure 4: MTAB-7902 (https://www.ebi.ac.uk/arrayexpress/experiments/E-MTAB-7902/), data linked to figure 7: GSE128050 (https://www.ncbi.nlm.nih.gov/geo/query/acc.cgi?acc=GSE128050). Expression data from young and old Neo1 mutant or control HSC can be found in supplemental table 1. Expression data from the analysis of young and old LSK-SLAM cells can be found in supplemental table 2, Source data for all 7 main figures and all 4 supplemantary figures is available in supplemental table 3. Nucleotide sequences and all other source data is available upon reasonable request from the corresponding author.

# Field-specific reporting

Please select the one below that is the best fit for your research. If you are not sure, read the appropriate sections before making your selection.

- [x] Life sciences
- [ ] Behavioural & social sciences
- [ ] Ecological, evolutionary & environmental sciences

For a reference copy of the document with all sections, see nature.com/documents/nr-reporting-summary-flat.pdf

# Life sciences study design

All studies must disclose on these points even when the disclosure is negative.

| | |
|---|---|
| Sample size | Sample size was determined based on extensive experience with similar experiments in our laboratory (Cabezas-Wallscheid et al., 2014 and 2017, Wilson et al. 2008). |
| Data exclusions | Sample exclusion was done only as a result of premature mouse death or if clear errors in pre-processing occured (Figure 2e, 12w timepoint, Figure3d, 10+ 12 month timepoint). |
| Replication | All attempts of replication were successful. Key experiments were performed at least twice, exact number of independent experiments with various biological replicates can be found in the figure legends. |
| Randomization | All samples/ mice were analysed and allocated randomly. |
| Blinding | No blinding occured, as the experiments performed made it impossible to implement. |

# Reporting for specific materials, systems and methods

We require information from authors about some types of materials, experimental systems and methods used in many studies. Here, indicate whether each material, system or method listed is relevant to your study. If you are not sure if a list item applies to your research, read the appropriate section before selecting a response.

## Materials & experimental systems

| n/a | Involved in the study | |
|---|---|---|
| [ ] | [x] | Antibodies |
| [x] | [ ] | Eukaryotic cell lines |
| [x] | [ ] | Palaeontology |
| [ ] | [x] | Animals and other organisms |
| [x] | [ ] | Human research participants |
| [x] | [ ] | Clinical data |

## Methods

| n/a | Involved in the study | |
|---|---|---|
| [x] | [ ] | ChIP-seq |
| [ ] | [x] | Flow cytometry |
| [x] | [ ] | MRI-based neuroimaging |

## Antibodies

Antibodies used

anti-mouse CD4-PE-Cy7 (clone: GK1.5) eBioscience Cat#25-0041-82; RRID: AB_469576
anti-mouse CD8a-PE-Cy7 (clone: 53-6.7) eBioscience Cat#25-0081-81; RRID: AB_469583
anti-mouse CD11b-PE-Cy7 (clone: M1/70) eBioscience Cat#25-0112-82; RRID: AB_469588
anti-mouse B220-PE-Cy7 (clone: RA3-6B2) eBioscience Cat#25-0452-81; RRID: AB_469626
anti-mouse Ter119-PE-Cy7 (clone: TER-119) eBioscience Cat#25-5921-81; RRID: AB_469660
anti-mouse Gr1-PE-Cy7 (clone: RB6-8C5) eBioscience Cat#25-5931-82; RRID: AB_469663
anti-mouse CD117(c-Kit)-APC (clone: 2B8) eBioscience Cat#17-1171-82; RRID: AB_469430
anti-mouse Ly-6A/E(Sca-1)-APC-Cy7 (clone: D7) BD Biosciences Cat#560654; RRID: AB_1727552
anti-mouse CD150-PE-Cy5 (clone: TC15) BioLegend Cat#115912; RRID: AB_493598
anti-mouse CD48-PB (clone: HM48-1) BioLegend Cat#103418; RRID: AB_756140
anti-mouse CD135-PE (clone: A2F10) eBioscience Cat#12-1351-81; RRID: AB_465858
anti-mouse CD34-FITC (clone: RAM34) eBioscience Cat# 11-0341-82; RRID:AB_465021
anti-human Ki67-AF647 (clone: B56) BD Biosciences Cat#558615; RRID: AB_647130
BD APC BrdU Flow Kit BD Biosciences Cat#552598
anti-mouse CD45-FITC (clone: 30-F11) eBioscience Cat # 11-0451-82, RRID:AB_465050
anti-mouse CD45.1-PB (clone: A20) eBioscience Cat#25-0453-82; RRID: AB_469629
anti-mouse CD45.2-FITC (clone: 104) eBioscience Cat #11-0454-82; RRID:AB_465061
Anti-mouse CD127- PE (clone: SB/199) BioLegend Cat#121111; , RRID:AB_493510
Anti-mouse CD16/32- PE-cy7 (clone: 93) BioLegend Cat#101317, RRID:AB_2104157

Anti-GFP AF488 abcam Cat#ab192863, Lot:GR201295-1
anti-mouse Cdk6 (clone:K6.83) abcam Cat#ab77674, RRID:AB_1566039, Lot: 268274-4
anti-mouse Neo1(clone: RM0124-3G55) abcam Cat#ab86577; RRID:AB_1925240, Lot: GR137-230-1
anti-mouse Neo1-Biotin R&D Cat#BAF1079; RRID:AB_2251295
Anti-mouse Pdpn- APC (clone: 8.1.1) Biolegend Cat# 127409, RRID:AB_10612940
Anti-mouse CD31- BV421 (clone: 390) Biolegend Cat# 102424, RRID:AB_2650892

For flow cytometry antibodies, Lot numbers can't reasonably be provided, as multiple different lots have been used over the course of this study. Depending on the individual experimental setting flow cytometry antibodies from the same clone but coupled to different fluorochromes were used.

Validation

All flow cytometry antibodies were already established and commonly used in our laboratory and have been published multiple times by us and other groups. Primary Antibodies used for IF of NEO1 were validated on liver sections of Neo1gt/gt mice as previously described (Zhou et al.,2010, blood). CDK6 was validated on more vs. less quiescent cells as described in Cabezas et al., 2017, Cell).

## Animals and other organisms

Policy information about studies involving animals; ARRIVE guidelines recommended for reporting animal research

Laboratory animals

C57BL/6J background:SCL-tTA; H2B-GFP, Gprc5c-GFP, FUCCI, Neo1gt/gt, Ntn1ßgeo/+, Ntn1fl/fl, + / LSL-Rosa26-Ntn1 , CAGGS:CreERT2;Ntn1fl/fl, CAGGS:CreERT2;+ / LSL-Rosa26-Ntn1, SMA:CreERT2;Ntn1fl/fl, c-Myc-eGFP, p65-GFP and wildtype C57BL/6J, Ly5.1 or C57BL/6J;Ly5.1 mice.

BALB/C-background:
Sma-RFP mice
For all experiments age and sex matched littermate controlls were used. Male and female animals were used. Mice were between 5- weeks to 30 months of age, depending on the respective experiments.

Wild animals

No wild animals were used in this study

Field-collected samples

No field collected samples were used in this study

Ethics oversight

All experiments were approved by the Regierungspräsidium Karlsruhe, Animal Care and Use Committee of Albert Einstein College of Medicine, the Instantie voor Dierenwelzijn (IvD) committee, Universitair Medisch Centrum Groningen/Rijksuniversiteit Groningen or University of Lyon local Animal Ethic Evaluation Committee

Note that full information on the approval of the study protocol must also be provided in the manuscript.

## Flow Cytometry

### Plots

Confirm that:

- [x] The axis labels state the marker and fluorochrome used (e.g. CD4-FITC).
- [x] The axis scales are clearly visible. Include numbers along axes only for bottom left plot of group (a 'group' is an analysis of identical markers).
- [x] All plots are contour plots with outliers or pseudocolor plots.
- [x] A numerical value for number of cells or percentage (with statistics) is provided.

### Methodology

Sample preparation

Briefly, BM was isolated from pooled femora, tibiae, ilia and vertebrae by gentle crushing in PBS using a mortar and pistil. If no depletion of lineage-positive cells was performed, lysis of erythrocytes was performed using ACK Lysing Buffer (Thermo Fisher Scientific). To deplete lineage-positive cells for experiments involving cell- sorting, we used the Dynabeads Untouched Mouse CD4 Cells Kit (Invitrogen). Briefly, total BM was stained for 30 min with 100µl / mouse of the Lineage Cocktail provided in the Dynabeads Untouched Mouse CD4 Cells Kit (Invitrogen) in PBS. Labelled cells were then incubated for 20 min with 1.5 ml / mouse of washed polyclonal sheep anti-rat IgG coated Dynabeads provided in the Kit. Cells were depleted using a magnet, enriching for the lineage-negative (Lineage-) cell fraction. To purify HSC and MPP1-4, the Lineage- fraction was stained for 30 min

Instrument

For cell sorting:
FACS Aria I, II and III, FACS Aria Fusion (Becton Dickinson) or MoFlo Astrios or XDP cell sorters (Beckman Coulter)
For analysis:
LSRII, LSR Fortessa

Software

Analysis performed with FlowJo, Versions 6-10.5.3, statistical analysis with Graphpad Prism, versions 6-8.1.2

Cell population abundance

Cell frequencies and behaviour, e.g. cell cycle status were in line with published data by our lab and others. Commonly known phenomena like expansion of the HSC compartment upon ageing or myeloid bias were recapitulated. Because of the rarity of the

sorted populations, we did noat perform resorts with every experiments, but 1 hours after in vitro culture, >80% of HSC still harboured the correct immunophenotype.

Gating strategy

HSC gating:
FSC-A v. SSC-A (cell gate excluding debris) --> FSC-A v. FSC-H (doublet exclusion)--> FSC-A v. Lineage (B220, CD11b, CD4, CD8,, Gr1, Ter119 e.g. PE-cy7) (Gating on lineage negative) --> cKIT (APC) v. SCA-1 (APC-cy7) (gating on double pos cells) --> CD150 (PE-Cy5) v. CD48 (PB) (gating on CD150+, CD48-) --> CD150 (PE-Cy5) v. CD34 (FITC)

for transplantations another step with gating on CD45.1 (PB) v. CD45.2 (FITC) was added and fluorochromes were adjusted.
for cell cycle analysis, Ki67 (PE-Cy7) v. DAPi staining was added and fluorochromes were adjusted.
for BrdU analysis, anti-BrdU (APC) was added and fluorochromes were adjusted.
for use of GFP+ or FUCCI mice, fluorochromes were adjusted.
for analysis of dormant HSC and isolation of RNA from MPP4 and MPP3 CD135 (PE) was added as an additional marker and fluorochromes were adjusted.

Differentiated cells:
FSC-A v. SSC-A (cell gate excluding debris) --> FSC-A v. FSC-H (doublet exclusion) --> CD45.1 (PB) v. CD45.2 (FITC) ( gated on CD45.2+ ) --> CD11b/Gr1/CD4/CD8 (PE) v. CD11b/Gr1/B220 (APC)

For AEC/SEC/SMA-RFP-mice:
FSC-A v. SSC-A (cell gate excluding debris) --> FSC-A v. FSC-H (doublet exclusion)--> CD45 (FITC) v. CD31 (PB) (gating on CD31+, CD45-) --> SCA-1 (APC-cy7) v. PDPN (APC) isolation of AEC/SEC OR pregating on CD45-/CD31- --> gating on FSC-A v. RFP isolation of RFP+ stromal cells.

☒ Tick this box to confirm that a figure exemplifying the gating strategy is provided in the Supplementary Information.
