# Supplementary material for: Niche derived netrin-1 regulates hematopoietic stem cell dormancy via its receptor neogenin-1
Source: Nat Commun. 2021 Jan 27;12:608. doi: 10.1038/s41467-020-20801-0 (PMC7840807; doi:10.1038/s41467-020-20801-0)
Supplement: Supplementary file 3 — Description of Additional Supplementary Files [file 41467_2020_20801_MOESM3_ESM.pdf]

Description of Additional Supplementary Files

File Name: Supplemental Data1:
Description: RNA-seq table containing relative expression values of Neo1 mutant vs. wildtype HSCs

File Name: Supplementary Data 2:
Description: RNA-seq table containing relative expression values of young vs. old HSCs

File Name: Supplementary Data 3:
Description: Source data table used for generation of plots in this manuscript
